# Pilot study: radiomic analysis for predicting treatment response to whole-brain radiotherapy combined temozolomide in lung cancer brain metastases

**DOI:** 10.3389/fonc.2024.1395313

**Published:** 2024-08-13

**Authors:** Yichu Sun, Fei Liang, Jing Yang, Yong Liu, Ziqiang Shen, Chong Zhou, Youyou Xia

**Affiliations:** ^1^ Department of Radiation Oncology, The First People's Hospital of Lianyungang/Lianyungang Clinical College of Nanjing Medical University, Lianyungang, Jiangsu, China; ^2^ Department of Radiation Oncology, The Affiliated Lianyungang Hospital of Xuzhou Medical University/The First People's Hospital of Lianyungang, Lianyungang, Jiangsu, China; ^3^ Department of Radiation Oncology, Xuzhou Central Hospital, Xuzhou, Jiangsu, China

**Keywords:** brain metastases, whole-brain radiation therapy, temozolomide, radiomics, nomogram

## Abstract

**Objective:**

The objective of this study is to assess the viability of utilizing radiomics for predicting the treatment response of lung cancer brain metastases (LCBM) to whole-brain radiotherapy (WBRT) combined with temozolomide (TMZ).

**Methods:**

Fifty-three patients diagnosed with LCBM and undergoing WBRT combined with TMZ were enrolled. Patients were divided into responsive and non-responsive groups based on the RANO-BM criteria. Radiomic features were extracted from contrast-enhanced the whole brain tissue CT images. Feature selection was performed using t-tests, Pearson correlation coefficients, and Least Absolute Shrinkage And Selection (LASSO) regression. Logistic regression was employed to construct the radiomics model, which was then integrated with clinical data to develop the nomogram model. Model performance was evaluated using receiver operating characteristic (ROC) curves, and clinical utility was assessed using decision curve analysis (DCA).

**Results:**

A total of 1834 radiomic features were extracted from each patient's images, and 3 features with predictive value were selected. Both the radiomics and nomogram models exhibited satisfactory predictive performance and clinical utility, with the nomogram model demonstrating superior predictive value. The ROC analysis revealed that the AUC of the radiomics model in the training and testing sets were 0.776 and 0.767, respectively, while the AUC of the nomogram model were 0.799 and 0.833, respectively. DCA curves demonstrated that both models provided benefits to patients across various thresholds.

**Conclusion:**

Radiomic-defined image biomarkers can effectively predict the treatment response of WBRT combined with TMZ in patients with LCBM, offering potential to optimize treatment decisions for this condition.

## Introduction

Lung cancer (LC) ranks among the most prevalent malignant tumors globally, accounting for 11.4% of all cancer incidences and contributing to an 18% mortality rate (1). Brain metastasis (BM), a common form of metastasis in LC, poses a formidable challenge in the treatment and disease management due to its significant threat to patients' functional independence and survival, with a median survival period ranging from 4 to 10 months post-treatment (2). The unique anatomical and physiological characteristics of the brain present challenges in finding effective treatment strategies for BM. Locally, whole-brain radiotherapy (WBRT) remains a pivotal local therapeutic modality for BM, primarily targeting patients with multiple brain metastases who are unsuitable for stereotactic radiosurgery (SRS) or surgical intervention. However, the central nervous system's limited tolerance to radiation restricts the dose of WBRT, making tumor reduction challenging. Systemically, targeted tyrosine kinase inhibitors are applicable only to a minority of patients with specific driver gene mutations. Platinum-based chemotherapy regimens and immune checkpoint inhibitors constitute the mainstay of systemic therapy for advanced LC; however, their efficacy against BM is limited due to the blood-brain barrier (3).

Temozolomide (TMZ), a novel imidazotetrazine alkylating agent, readily crosses the blood-brain barrier to exert intracranial anti-tumor effects, thereby enhancing the local efficacy or survival benefits of radiotherapy for brain metastases (4, 5). A Chinese treatment guideline for lung cancer brain metastases (LCBM) recommends TMZ as a radiosensitizer in combination with WBRT, suggesting it as a promising alternative (6). In a moderately sized real-world study, the addition of TMZ to WBRT increased intracranial objective response rates from 20.2% to 34.9%, disease control rates from 92.7% to 98.4%, progression-free survival from 4.9 to 5.9 months, and overall survival from 5.9 to 8.5 months (7). Nevertheless, a recent meta-analysis incorporating 25 prospective studies indicated that the addition of TMZ to WBRT significantly increased objective response rates while also elevating the risk of hematological and gastrointestinal toxicities (8). This combination therapy is not suitable for every individual. Therefore, developing tools to predict treatment response can facilitate individualized medical decisions, avoid ineffective treatments, and prevent treatment toxicity. However, there is currently a lack of reported predictive model studies for this patient population.

Radiomics, an emerging field that integrates artificial intelligence image recognition with medical imaging technology, extracts high-throughput omics data with biological significance from medical images through a series of standardized quantitative calculation methods. After appropriate feature selection and machine learning methods, these imaging biomarkers can reflect the potential differences and complexities of diseases (9, 10). In recent years, some studies have successfully applied radiomics to predict the microscopic heterogeneity of lung cancer brain metastases, such as pathological subtypes (11), PD-L1 expression (12), and EGFR mutation status (13). Thus, radiomics holds significant potential in distinguishing individual differences in LCBM, perhaps providing a potential pathway for predicting the efficacy of WBRT combined with TMZ.

Therefore, this study aimed to explore the feasibility and application value of radiomics technology in predicting the efficacy of WBRT combined with TMZ for LCBM, expected to optimize personalized treatment decisions.

## Materials and methods

### Patients recruiting

The medical center's ethics committee approved this retrospective study (Permit No. LW-20240119001-01) with waived written informed consent from patients. Clinicopathological data were retrieved by two radiation oncologists through review of medical records and telephone follow-ups, and then evaluated by a senior radiation oncology expert. Between January 2018 and June 2023, a total of 78 patients with LCBM who underwent WBRT combined with TMZ at our radiotherapy center were identified. After screening based on inclusion and exclusion criteria, 53 patients were included in this study and randomly divided into training and testing sets at an 8:2 ratio. Inclusion criteria were as follows: (1) Definite diagnosis of LCBM through histopathology or typical CT or MRI findings; (2) Available contrast-enhanced whole-brain CT and Eclipse treatment planning system images; (3) Completed WBRT synchronized with temozolomide treatment as planned and evaluated for efficacy. Exclusion criteria were: (1) Presence of intracranial organic diseases or history of intracranial radiotherapy; (2) Occurrence of acute cerebrovascular events during treatment; (3) Incomplete data retrieval despite review of medical records and telephone follow-ups. WBRT was delivered using one of the two American Varian linear accelerators at our radiotherapy center, with a planned target volume (PTV) expansion of 0.3cm beyond the whole brain. The radiotherapy doses were delivered at 30 Gy in 10 fractions, 37.5 Gy in 15 fractions, or 40 Gy in 20 fractions, in accordance with the NCCN guidelines (14). Radiation therapy was administered five times per week, along with concurrent oral or intravenous administration of temozolomide at a dosage of 75-100 mg/m²/day. Treatment response assessment was conducted at 1.5 to 2 months post-treatment, following the Response Assessment in Neuro-Oncology Brain Metastases (RANO-BM) criteria (15). Patients with complete response (CR) or partial response (PR) were divided into responsive group, while those with stable disease (SD) or disease progression (PD) were divided into non-responsive group.

### Image acquisition

All contrast-enhanced CT scans were performed using a Siemens SOMATOM Definition AS+ 64-slice CT simulator at our radiotherapy center, covering from the cranial vertex to the lower boundary of the neck, with a slice thickness of 3 mm. The final WBRT treatment plan was devised and confirmed by the radiation oncology expert team at our institution. DICOM-format whole-brain CT image files and RT-structure files were exported via the Eclipse system and ARIA network workstation. Subsequently, we utilized the OnekeyAI platform (www.medai.icu/) to parse and convert the DICOM files and RT-structure files into NIFTI-format localization CT images and corresponding radiotherapy target volume. The definition of regions of interest (ROIs) in radiomics primarily relied on lesion-based or anatomy-based criteria (16, 17). Given the scope of local radiotherapy across the entire brain in this study, ROIs were defined in 3D based on the anatomical structures of the whole brain tissue. The final ROIs were manually adjusted by one radiation oncologist using 3dslicer [5.1.0] software (18) and verified by a senior radiation oncology expert.

### Radiomics features extraction

Image preprocessing and feature extraction were conducted using the PyRadiomics [3.0.1] Python package. Comprehensive image preprocessing included standardizing the image to a range of 0-1000 Hounsfield Units (HU) and spatially resampling the image to a consistent voxel size of 3mm x 3mm x 3mm. Various filters were applied to enhance the images, including Gaussian Laplacian (LoG) filter, wavelet transformation, Local Binary Patterns in 3D (LBP3D), as well as mathematical transformations such as exponential, square, square root, and logarithmic transformations to highlight different features and textures in the images. Subsequently, shape features, first-order statistical features, and texture features were extracted from the preprocessed images and the transformed images obtained from the aforementioned filters. The texture features included Gray Level Co-occurrence Matrix (GLCM), Gray Level Dependence Matrix (GLDM), Gray Level Run Length Matrix (GLRLM), Gray Level Size Zone Matrix (GLSZM), and Neighboring Gray Tone Difference Matrix (NGTDM). Further descriptions of the feature extraction process and the YAML configuration file used with pyradiomics were provided in **Supplementary File 1**. All feature definitions and calculation methods adhered to the standards of the Imaging Biomarker Standardization Initiative (IBSI) (10).

### Feature selection

All radiomic features were transformed using the formula Z=(X-μ)/σ , and then the feature selection process commenced in the training dataset. Initially, the most significantly distinguishing features between the responsive and non-responsive groups were identified through statistical analysis using the t-test, with only features with p < 0.01 retained. Subsequently, redundant features were removed using Pearson correlation coefficients and greedy recursive feature elimination. When any two features had a correlation coefficient exceeding 0.9, one of them was systematically eliminated. In each iteration, the 10 most redundant features with the highest correlation coefficients were discarded. Finally, the Absolute Shrinkage and Selection (LASSO) model was developed, and the lambda parameter was determined through 10-fold cross-validation, aiming to minimize the mean squared error (MSE). The non-zero coefficient features were identified at that lambda.

### Radiomics signature

The final features selected by the Lasso model were used to construct the radiomics prediction model. Logistic regression (LR), one of the most commonly used machine learning algorithms in medical data, was chosen for this purpose. The output results of the prediction model were used to construct the radiomic signature. Subsequently, confusion matrices and receiver operating characteristic (ROC) curves were employed for both the training and testing sets to demonstrate predictive performance. Additionally, decision curve analysis (DCA) was conducted to assess clinical utility across different thresholds. Furthermore, the model output results were utilized to generate radiomics signature, serving as biomarkers for subsequent analysis.

### Nomogram model

**Figure 1:**
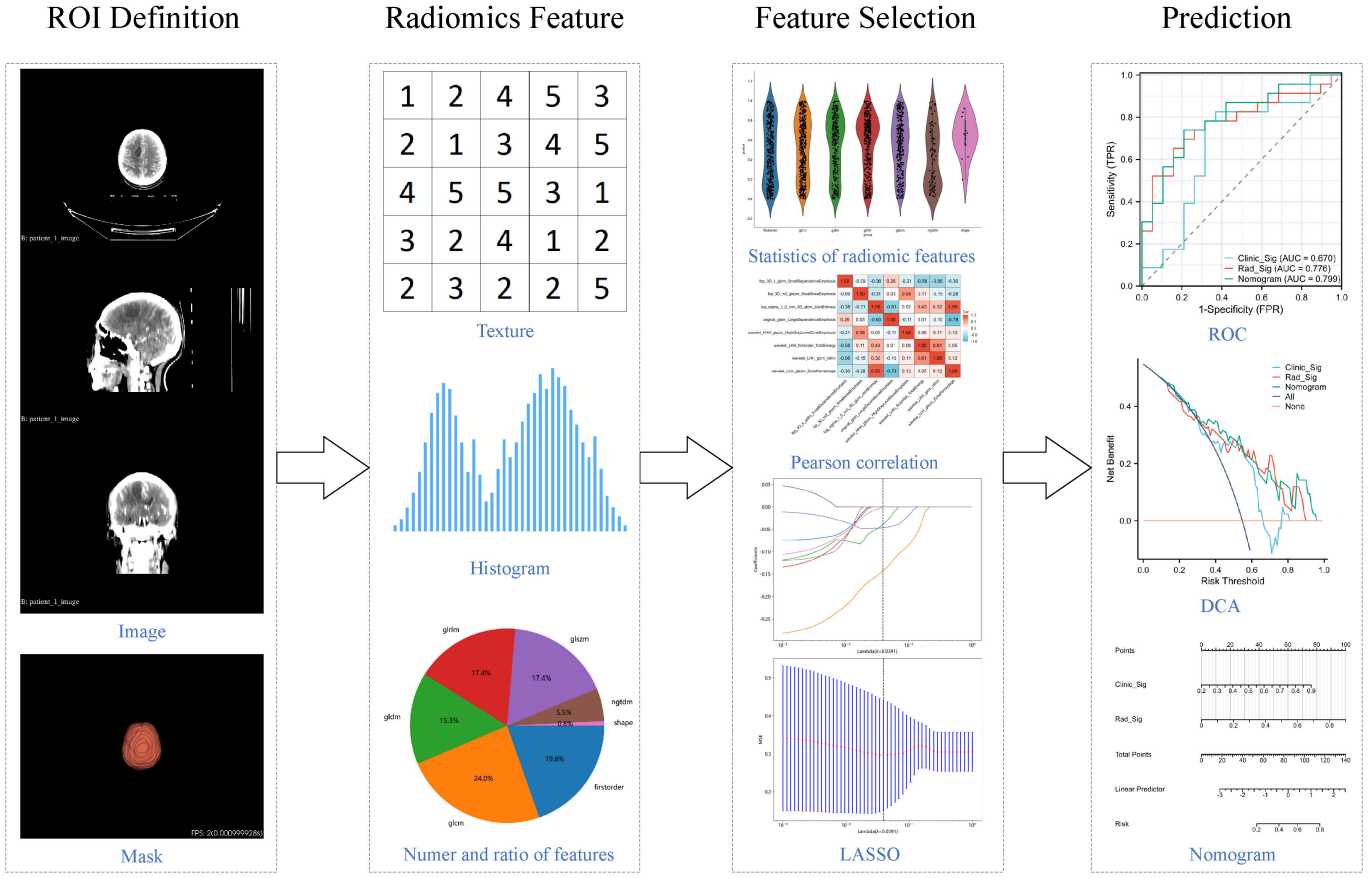
Workflow of radiomics analysis in this study.

We also explored the potential enhancement of the radiomics model's predictive performance by incorporating clinical variables. Baseline features including age, gender, Karnofsky Performance Status (KPS), serum carcinoembryonic antigen (CEA) levels, and serum neuron-specific enolase (NSE) were used to generate clinical signature through the same LR results, ensuring its comparability to radiomics signature. Subsequently, the nomogram model was developed by integrating clinical signature and radiomics signature. ROC curves were generated for both the training and testing sets to evaluate and compare their performance. DCA curves were employed to evaluate and compare their clinical utility across different thresholds. The comprehensive workflow of the radiomics analysis is depicted in **Figure 1**.

### Statistics and analysis

Normality was assessed using the Shapiro-Wilk test. Continuous variables with normal distribution were presented as mean and standard deviation and analyzed using the t-test. Continuous variables with severe skewed distribution were presented as median and interquartile range and analyzed using the Wilcoxon test. Categorical variables were presented as frequency and percentage and analyzed using the chi-square test or chi-square test with Yates' correction. The significance level for hypothesis testing was set at 0.05. Statistical analysis of baseline data was performed using the stats [4.2.11] R package, nomogram analysis using the rms [6.4.0] R package, ROC analysis using the pROC [1.18.0] R package, DCA analysis using the rmda [1.6] R package, and results were visualized using the ggplot2 [3.3.6] R package. Model building was conducted using the scikit-learn [1.0.2] Python package.

## Results

### Patient’s characteristics

The clinical baseline characteristics presented in **Table 1** encompassed a total of 53 patients with LCBM, with 33 cases (62.3%) of non-small cell lung cancer and 20 cases (37.7%) of small cell lung cancer. Among these individuals, there were 17 females (32.1%) and 36 males (67.9%), with ages ranging from 36 to 83 years and a mean age of 60.4 years. According to the evaluation results, there were 24 cases in the responsive group and 29 cases in the non-responsive group. Statistical analysis revealed no significant differences in all clinical variables between the training and testing sets, indicating the effectiveness of the random dataset partition.

**Table 1:** Baseline characteristics of patients in the training and validation sets.

Characteristics	Overall	Training	Test	pvalue
n	53	42	11	
Age, mean ± sd	60.4 ± 9.3	60.8 ± 9.9	58.9 ± 6.3	0.552
CEA, median (IQR)	6.8 (3.0, 64.5)	7.8 (3.4, 65.1)	2.9 (2.2, 34.6)	0.139
NSE, median (IQR)	19.9(14.5, 29.6)	18.9 (14.6, 26.8)	27.0 (18.8, 31.4)	0.303
Gender, n (%)				0.999
Female	17 (32.1%)	13 (24.5%)	4 (7.5%)	
Male	36 (67.9%)	29 (54.7%)	7 (13.2%)	
KPS, n (%)				0.922
≥70	32 (60.4%)	26 (49.1%)	6 (11.3%)	
<70	21 (39.6%)	16 (30.2%)	5 (9.4%)	
Pathology, n (%)				0.649
NSCLC	33 (62.3%)	25 (47.2%)	8 (15.1%)	
SCLC	20 (37.7%)	17 (32.1%)	3 (5.7%)	
BM_Number, n (%)				0.999
<5	24 (45.3%)	19 (35.8%)	5 (9.4%)	
≥5	29 (54.7%)	23 (43.4%)	6 (11.3%)	
CNS_symptom, n (%)				0.999
No	23 (43.4%)	18 (34.0%)	5 (9.4%)	
Yes	30 (56.6%)	24 (45.3%)	6 (11.3%)	
PTV_dose_cGy, n (%)				0.807
3000	35 (66.0%)	27 (50.9%)	8 (15.1%)	
3750	8 (15.1%)	8 (15.1%)	2 (3.8%)	
4000	10 (18.9%)	7 (13.2%)	1 (1.9%)	
TMZ_route, n (%)				0.552
Intravenous	32 (60.4%)	24 (45.3%)	8 (15.1%)	
Oral	21 (39.6%)	18 (34%)	3 (5.7%)	

IQR, interquartile range; sd, standard deviation; NSCLC, non-small cell lung cancer; SCLC, small cell lung cancer; CEA, carcinoembryonic antigen; NSE, neuron-specific enolase; CNS, central nervous system; TMZ, temozolomide.

### Radiomics features

**Figure 2:**
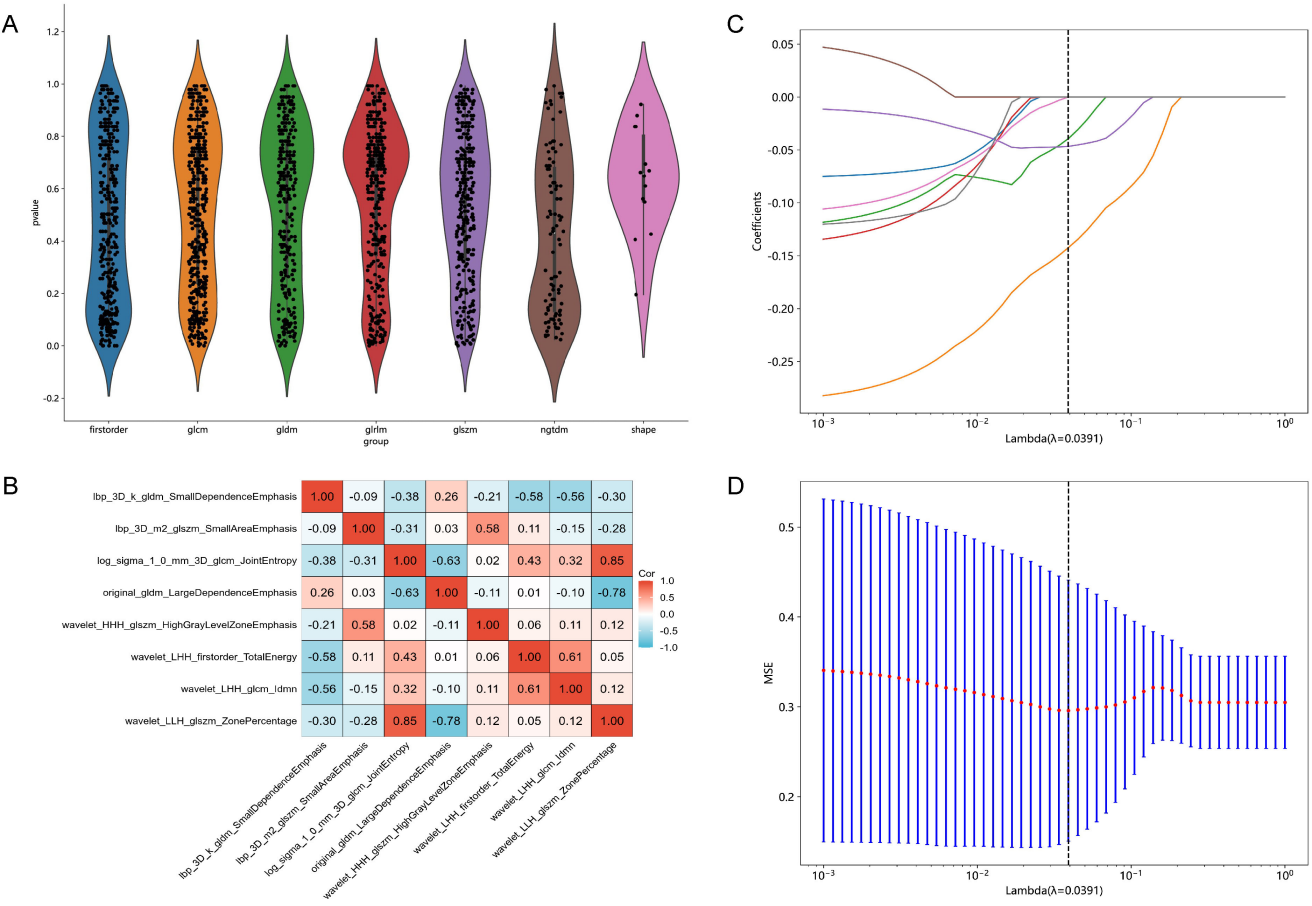
Results of feature selection. **(A)** Violin plot illustrating the distribution of p-values from the statistical tests of all radiomic features. **(B)** Heatmap showing the correlation coefficients of the 8 selected features obtained through Pearson correlation coefficients combined with a greedy strategy. **(C)** Variations in the weight coefficients of the 8 radiomic features with lambda during the 10-fold cross-validation process of the least absolute shrinkage and selection operator (LASSO) model. **(D)** The change in mean squared error (MSE) with lambda during the 10-fold cross-validation process of the LASSO model.

Each patient's CT images produced 1834 radiomic features. Detailed results can be found in **Supplementary File 1**. Initially, 53 features with p < 0.01 were identified through t-test analysis, as depicted in **Figure 2A**. Subsequently, 8 features were selected by the last iteration of greedy recursive elimination. Their correlation coefficients were presented in **Figure 2B**. Finally, during the 10-fold cross-validation process of the LASSO model, the weight coefficients of the 8 radiomic features varied with lambda, as shown in **Figure 2C**, while the change in MSE with lambda was illustrated in **Figure 2D**. The lambda parameter associated with the minimum MSE was found to be 0.0391, corresponding to the retention of 3 non-zero coefficient features in the model: "lbp_3D_m2_glszm_SmallAreaEmphasis", "log_sigma_1_0_mm_3D_glcm_JointEntropy", and "wavelet_HHH_glszm_HighGrayLevelZoneEmphasis".

### Predictive performance

**Figure 3:**
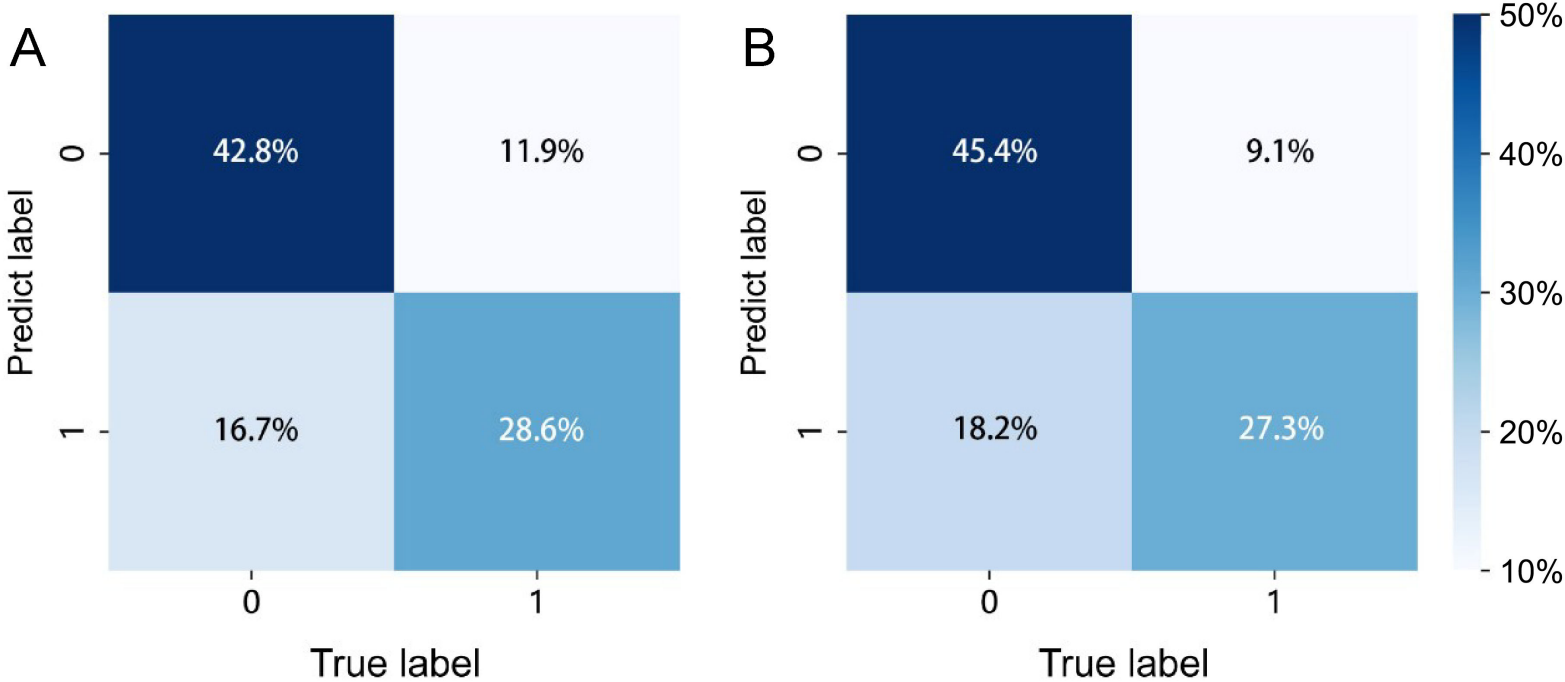
The prediction confusion matrices of the radiomics model in the training set **(A)** and testing set **(B)**.

**Figure 4:**
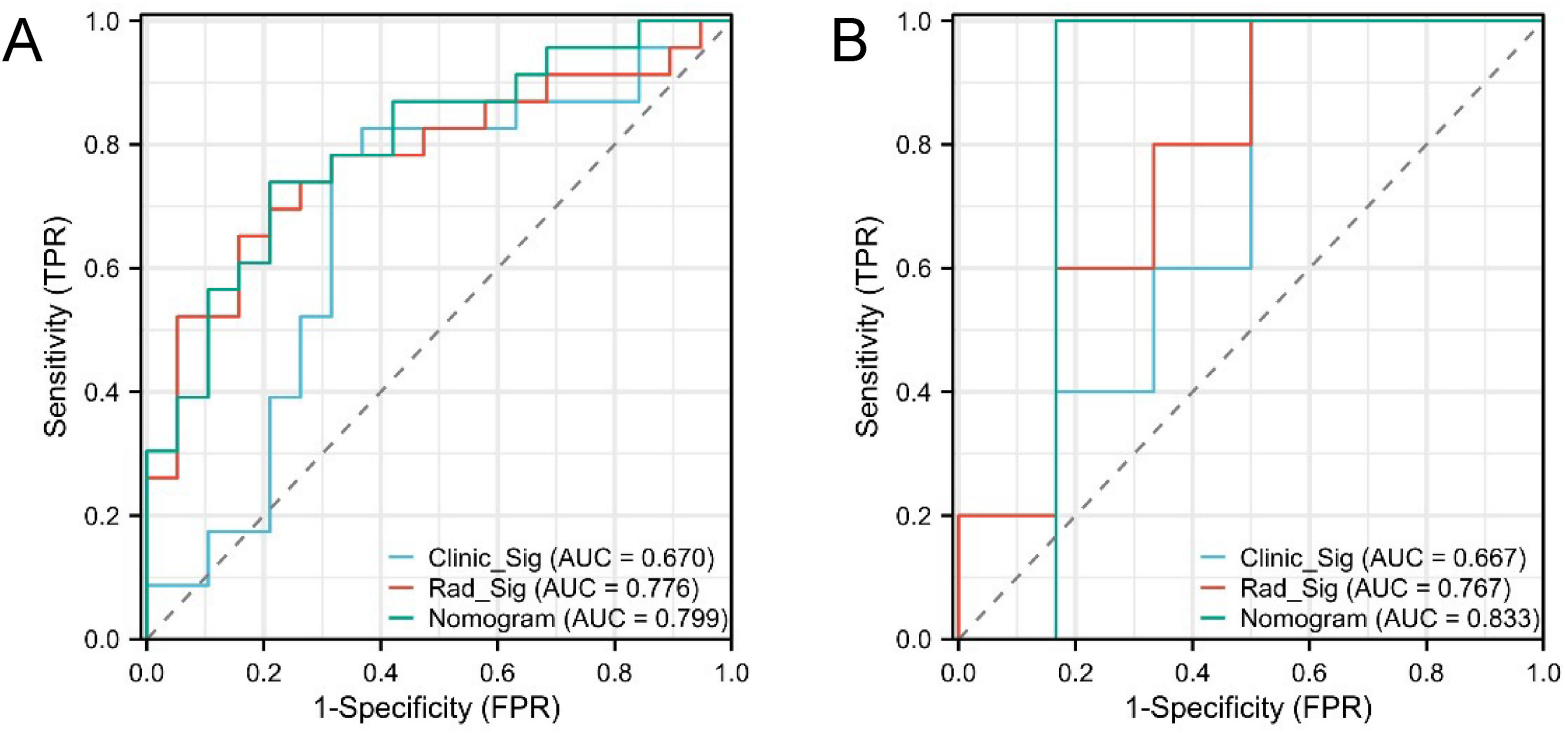
ROC curves of the three models in the training set **(A)** and testing set **(B)**. Both radiomics signature (Rad_Sig) and nomogram exhibited AUC values exceeding 0.75, indicating good predictive performance.

**Figure 5:**
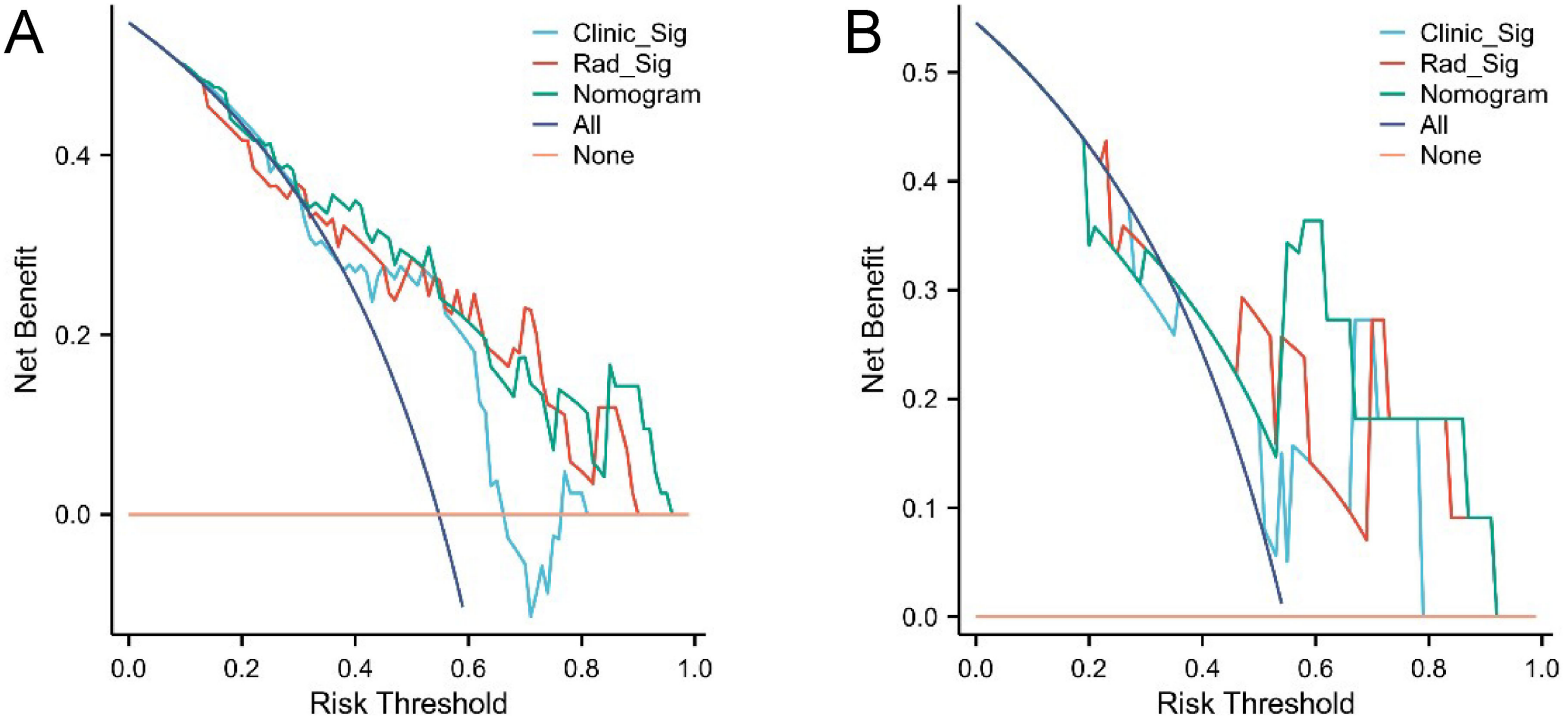
DCA curves of the three models in the training set **(A)** and testing set **(B)**. Radiomics signature (Rad_Sig) and nomogram demonstrated net benefits to patients across the majority of risk thresholds.

**Figure 6:**
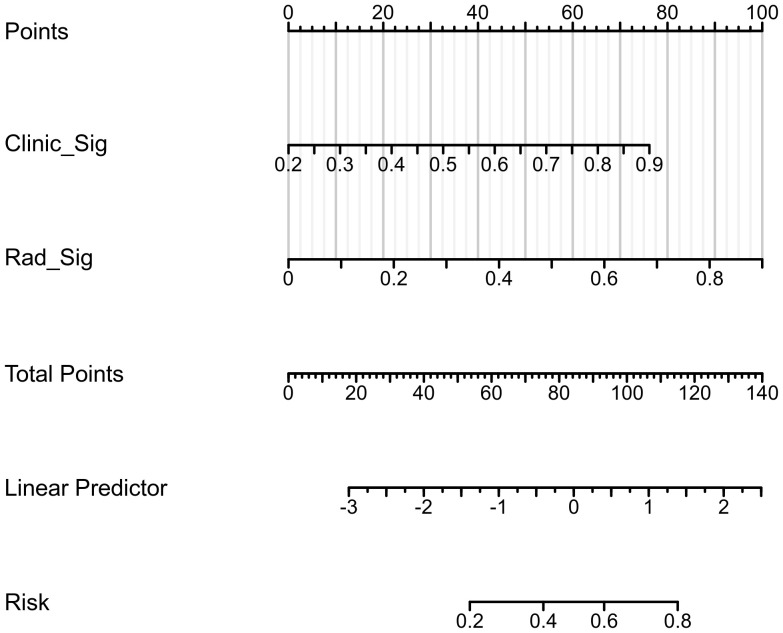
The visualization of the nomogram model.

The confusion matrices for the radiomics model in the training and testing sets were displayed in **Figures 3A, B**, respectively, with prediction accuracies of 71.4% and 72.7%. The ROC curves of the radiomics signature, clinical signature, and nomogram models on the training set were illustrated in **Figure 4A**, while those on the testing set were presented in **Figure 4B**. The AUC values on the training and testing sets were as follows: clinical signature: 0.670, 0.667; radiomics signature: 0.776, 0.767; nomogram: 0.799, 0.833. There were no indications of overfitting detected in any of the models. Both radiomics signature and nomogram showed AUC values surpassing 0.75, suggesting robust predictive performance. In contrast, clinical signature displayed AUC values below 0.7. The DCA curves on the training set were depicted in **Figure 5A**, while those on the testing set were shown in **Figure 5B**. Across various risk thresholds, radiomics signature and nomogram yielded net benefits to patients in most scenarios, outperforming clinical signature. Combining the results of ROC curves and DCA analysis, the nomogram surpassed radiomics signature in performance on both the training and testing sets. The visualization of the nomogram model was portrayed in **Figure 6**, illustrating the relationships between the nomogram and radiomics signature, as well as clinical signature.

## Discussion

This study attempted to utilize radiomic analysis to predict the treatment response of WBRT combined with TMZ for LCBM, demonstrating its feasibility and application value. Furthermore, integrating clinical information into the radiomic model through a nomogram can further improved predictive performance.

Radiomic technology aims to identify valuable biomarkers by extracting quantitative features from images, holding great potential for predicting outcomes in LC treatment (19–21). However, in studies on BM, researchers often focus on using radiomics to predict the efficacy of SRS (22–24), while research involving the prediction of WBRT treatment response is scarce. WBRT, as a crucial local treatment modality for BM, often faces challenges in reducing tumor size due to dose limitations on brain tissue (25). Data from a phase III clinical trial (PCI-P120-9801) suggest a correlation between tumor shrinkage after WBRT and improved survival and neurological function (26). Therefore, the development of tools to predict WBRT treatment response in patients with BM is crucial for making informed clinical decisions. Wang et al.'s study attempted to use radiomic technology to predict the treatment response of BM patients to WBRT, demonstrating excellent predictive performance. The AUC of the radiomic model was 0.928 and 0.837 in the training and testing sets, respectively, while the AUC of the clinical model was 0.650 and 0.598. The nomogram model combining clinical factors and radiomics achieved AUCs of 0.928 and 0.851 (27). Previous studies have also used radiomics to predict the efficacy of TMZ, but in the context of glioblastoma. For example, Li et al.'s study analyzed radiomic features and predicted efficacy in 53 patients with malignant glioblastoma receiving anlotinb and TMZ combination therapy, achieving AUCs above 0.9 in both the training and testing sets. However, this study did not incorporate clinical information into the radiomic model (28). Therefore, it is necessary to investigate the feasibility of using radiomics to predict the response of BM to WBRT combined with TMZ.

In this study, we aimed to reduce the heterogeneity among BM patients by focusing on those with primary LC. We gathered contrast-enhanced CT scans from BM patients who underwent WBRT in combination with TMZ at our academic medical center over the last five years for radiomic analysis. During feature selection, we pinpointed three predictive features using the LASSO model with the minimum mean squared error (MSE), all of which were related to filtered enhancement and transformed grayscale texture features. Notably, these features did not encompass shape or first-order statistical features, likely due to our ROIs definition based on anatomical structures, given the whole-brain scope therapy of our study. This ROIs strategy is advantageous as it incorporates not only intra-tumoral information but also data from the tumor periphery and surrounding normal tissue, all of which hold predictive potential in radiomics (16, 17, 29). Subsequently, we constructed predictive models and assessed their predictive value using ROC and DCA analysis. In ROC analysis, the AUCs of radiomic model were 0.776 and 0.767 in the training and testing sets, respectively, while those of clinical model were 0.670 and 0.667. The nomogram, combining clinical signature and radiomic signature, achieved AUCs of 0.799 and 0.833. These results suggest the feasibility of utilizing radiomics to predict treatment response of WBRT combined with TMZ in BM patients. Notably, prior research focusing on LC biomarkers has underscored that clinical signature complement radiomic signature by providing valuable insights (30). In our study, while clinical signature alone failed to predict treatment response, it could offer potential information to radiomic signature, thereby enhancing predictive performance, which was consistent with the findings of Wang et al.'s study (27). Moreover, our DCA analysis further affirmed the utility of both the radiomic model and the nomogram for clinical decision-making. In summary, these results underscore the potential of radiomics as a supplementary clinical tool for predicting treatment response of WBRT combined with TMZ in LCBM, thus facilitating personalized treatment strategies.

There are several limitations in our study. Firstly, the small sample size and retrospective data collection might introduce bias. Secondly, the clarity of enhanced CT images is not as high as MRI, potentially impacting the accuracy of the radiomic model (31). Thirdly, the single-center division of the testing set may not adequately validate the model's stability. Future research will utilize multi-modal imaging data and acquire multi-center validation datasets to enhance the accuracy and generalizability of predictive models.

## Conclusion

Overall, this study successfully predicted the treatment response of LCBM undergoing WBRT combined with TMZ using radiomic-defined image biomarkers. These findings offer potential for optimizing treatment decisions for LCBM in clinical practice and laying the groundwork for further clinical application research.

## Data Availability

The data analyzed in this study is subject to the following licenses/restrictions: The original data presented in this article are partially uploaded in **Supplementary File 1**. Inquiries can be directed to the corresponding author with a reasonable request. Requests to access these datasets should be directed to Xia Youyou, xia.youyou@njmu.edu.cn.
