# Supplementary material for: Pilot study: radiomic analysis for predicting treatment response to whole-brain radiotherapy combined temozolomide in lung cancer brain metastases
Source: Front Oncol. 2024 Aug 13;14:1395313. doi: 10.3389/fonc.2024.1395313 (PMC11347322; doi:10.3389/fonc.2024.1395313)
Supplement: Supplementary file 1 [file Datasheet1.zip › Supplementary File 1/Descriptions of the feature extraction.docx]

The standard 13 independent 3D displacement vectors: (1,0,0), (0,1,0), (0,0,1), (1,1,0), (1,0,1), (0,1,1), (1,-1,0), (1,0,-1), (0,1,-1), (-1,1,0), (-1,0,1), (0,-1,1), (1,1,1).

Feature Generation Process:

We employed multiple image processing techniques such as Original, Laplacian of Gaussian (LoG), Wavelet, and others. For each image type, features were extracted from various feature classes, including shape features, first-order statistics, and GLCM. For instance, in the case of GLCM, we used the aforementioned 13 directions and extracted multiple texture features such as Contrast, Entropy, and Correlation.

Naming Convention:

The features were named according to the format "ImageType_FeatureClass_FeatureName".

For example:

exponential_firstorder_10Percentile: Represents the 10th percentile from first-order statistics extracted from the exponential filter image.

exponential_glcm_Contrast: Represents the contrast feature from the GLCM matrix of the exponential filter image.

gradient_firstorder_Mean: Represents the mean value from first-order statistics extracted from the gradient image.

lbp_3D_k_glcm_Correlation: Represents the correlation feature from the GLCM matrix of the 3D Local Binary Pattern (LBP) image.

This naming convention ensures that each feature's source and specific parameters are transparent and easily understood.

The 1834 radiomics feature names:

exponential_firstorder_10Percentile,

exponential_firstorder_90Percentile,

exponential_firstorder_Energy,

exponential_firstorder_Entropy,

exponential_firstorder_InterquartileRange,

exponential_firstorder_Kurtosis,

exponential_firstorder_Maximum,

exponential_firstorder_Mean,

exponential_firstorder_MeanAbsoluteDeviation,

exponential_firstorder_Median,

exponential_firstorder_Minimum,

exponential_firstorder_Range,

exponential_firstorder_RobustMeanAbsoluteDeviation,

exponential_firstorder_RootMeanSquared,

exponential_firstorder_Skewness,

exponential_firstorder_TotalEnergy,

exponential_firstorder_Uniformity,

exponential_firstorder_Variance,

exponential_glcm_Autocorrelation,

exponential_glcm_ClusterProminence,

exponential_glcm_ClusterShade,

exponential_glcm_ClusterTendency,

exponential_glcm_Contrast,

exponential_glcm_Correlation,

exponential_glcm_DifferenceAverage,

exponential_glcm_DifferenceEntropy,

exponential_glcm_DifferenceVariance,

exponential_glcm_Id,

exponential_glcm_Idm,

exponential_glcm_Idmn,

exponential_glcm_Idn,

exponential_glcm_Imc1,

exponential_glcm_Imc2,

exponential_glcm_InverseVariance,

exponential_glcm_JointAverage,

exponential_glcm_JointEnergy,

exponential_glcm_JointEntropy,

exponential_glcm_MaximumProbability,

exponential_glcm_SumEntropy,

exponential_glcm_SumSquares,

exponential_gldm_DependenceEntropy,

exponential_gldm_DependenceNonUniformity,

exponential_gldm_DependenceNonUniformityNormalized,

exponential_gldm_DependenceVariance,

exponential_gldm_GrayLevelNonUniformity,

exponential_gldm_GrayLevelVariance,

exponential_gldm_HighGrayLevelEmphasis,

exponential_gldm_LargeDependenceEmphasis,

exponential_gldm_LargeDependenceHighGrayLevelEmphasis,

exponential_gldm_LargeDependenceLowGrayLevelEmphasis,

exponential_gldm_LowGrayLevelEmphasis,

exponential_gldm_SmallDependenceEmphasis,

exponential_gldm_SmallDependenceHighGrayLevelEmphasis,

exponential_gldm_SmallDependenceLowGrayLevelEmphasis,

exponential_glrlm_GrayLevelNonUniformity,

exponential_glrlm_GrayLevelNonUniformityNormalized,

exponential_glrlm_GrayLevelVariance,

exponential_glrlm_HighGrayLevelRunEmphasis,

exponential_glrlm_LongRunEmphasis,

exponential_glrlm_LongRunHighGrayLevelEmphasis,

exponential_glrlm_LongRunLowGrayLevelEmphasis,

exponential_glrlm_LowGrayLevelRunEmphasis,

exponential_glrlm_RunEntropy,

exponential_glrlm_RunLengthNonUniformity,

exponential_glrlm_RunLengthNonUniformityNormalized,

exponential_glrlm_RunPercentage,

exponential_glrlm_RunVariance,

exponential_glrlm_ShortRunEmphasis,

exponential_glrlm_ShortRunHighGrayLevelEmphasis,

exponential_glrlm_ShortRunLowGrayLevelEmphasis,

exponential_glszm_GrayLevelNonUniformity,

exponential_glszm_GrayLevelNonUniformityNormalized,

exponential_glszm_GrayLevelVariance,

exponential_glszm_HighGrayLevelZoneEmphasis,

exponential_glszm_LargeAreaEmphasis,

exponential_glszm_LargeAreaHighGrayLevelEmphasis,

exponential_glszm_LargeAreaLowGrayLevelEmphasis,

exponential_glszm_LowGrayLevelZoneEmphasis,

exponential_glszm_SizeZoneNonUniformity,

exponential_glszm_SizeZoneNonUniformityNormalized,

exponential_glszm_SmallAreaEmphasis,

exponential_glszm_SmallAreaHighGrayLevelEmphasis,

exponential_glszm_SmallAreaLowGrayLevelEmphasis,

exponential_glszm_ZoneEntropy,

exponential_glszm_ZonePercentage,

exponential_glszm_ZoneVariance,

exponential_ngtdm_Busyness,

exponential_ngtdm_Coarseness,

exponential_ngtdm_Complexity,

exponential_ngtdm_Contrast,

exponential_ngtdm_Strength,

gradient_firstorder_10Percentile,

gradient_firstorder_90Percentile,

gradient_firstorder_Energy,

gradient_firstorder_Entropy,

gradient_firstorder_InterquartileRange,

gradient_firstorder_Kurtosis,

gradient_firstorder_Maximum,

gradient_firstorder_Mean,

gradient_firstorder_MeanAbsoluteDeviation,

gradient_firstorder_Median,

gradient_firstorder_Minimum,

gradient_firstorder_Range,

gradient_firstorder_RobustMeanAbsoluteDeviation,

gradient_firstorder_RootMeanSquared,

gradient_firstorder_Skewness,

gradient_firstorder_TotalEnergy,

gradient_firstorder_Uniformity,

gradient_firstorder_Variance,

gradient_glcm_Autocorrelation,

gradient_glcm_ClusterProminence,

gradient_glcm_ClusterShade,

gradient_glcm_ClusterTendency,

gradient_glcm_Contrast,

gradient_glcm_Correlation,

gradient_glcm_DifferenceAverage,

gradient_glcm_DifferenceEntropy,

gradient_glcm_DifferenceVariance,

gradient_glcm_Id,

gradient_glcm_Idm,

gradient_glcm_Idmn,

gradient_glcm_Idn,

gradient_glcm_Imc1,

gradient_glcm_Imc2,

gradient_glcm_InverseVariance,

gradient_glcm_JointAverage,

gradient_glcm_JointEnergy,

gradient_glcm_JointEntropy,

gradient_glcm_MaximumProbability,

gradient_glcm_SumEntropy,

gradient_glcm_SumSquares,

gradient_gldm_DependenceEntropy,

gradient_gldm_DependenceNonUniformity,

gradient_gldm_DependenceNonUniformityNormalized,

gradient_gldm_DependenceVariance,

gradient_gldm_GrayLevelNonUniformity,

gradient_gldm_GrayLevelVariance,

gradient_gldm_HighGrayLevelEmphasis,

gradient_gldm_LargeDependenceEmphasis,

gradient_gldm_LargeDependenceHighGrayLevelEmphasis,

gradient_gldm_LargeDependenceLowGrayLevelEmphasis,

gradient_gldm_LowGrayLevelEmphasis,

gradient_gldm_SmallDependenceEmphasis,

gradient_gldm_SmallDependenceHighGrayLevelEmphasis,

gradient_gldm_SmallDependenceLowGrayLevelEmphasis,

gradient_glrlm_GrayLevelNonUniformity,

gradient_glrlm_GrayLevelNonUniformityNormalized,

gradient_glrlm_GrayLevelVariance,

gradient_glrlm_HighGrayLevelRunEmphasis,

gradient_glrlm_LongRunEmphasis,

gradient_glrlm_LongRunHighGrayLevelEmphasis,

gradient_glrlm_LongRunLowGrayLevelEmphasis,

gradient_glrlm_LowGrayLevelRunEmphasis,

gradient_glrlm_RunEntropy,

gradient_glrlm_RunLengthNonUniformity,

gradient_glrlm_RunLengthNonUniformityNormalized,

gradient_glrlm_RunPercentage,

gradient_glrlm_RunVariance,

gradient_glrlm_ShortRunEmphasis,

gradient_glrlm_ShortRunHighGrayLevelEmphasis,

gradient_glrlm_ShortRunLowGrayLevelEmphasis,

gradient_glszm_GrayLevelNonUniformity,

gradient_glszm_GrayLevelNonUniformityNormalized,

gradient_glszm_GrayLevelVariance,

gradient_glszm_HighGrayLevelZoneEmphasis,

gradient_glszm_LargeAreaEmphasis,

gradient_glszm_LargeAreaHighGrayLevelEmphasis,

gradient_glszm_LargeAreaLowGrayLevelEmphasis,

gradient_glszm_LowGrayLevelZoneEmphasis,

gradient_glszm_SizeZoneNonUniformity,

gradient_glszm_SizeZoneNonUniformityNormalized,

gradient_glszm_SmallAreaEmphasis,

gradient_glszm_SmallAreaHighGrayLevelEmphasis,

gradient_glszm_SmallAreaLowGrayLevelEmphasis,

gradient_glszm_ZoneEntropy,

gradient_glszm_ZonePercentage,

gradient_glszm_ZoneVariance,

gradient_ngtdm_Busyness,

gradient_ngtdm_Coarseness,

gradient_ngtdm_Complexity,

gradient_ngtdm_Contrast,

gradient_ngtdm_Strength,

lbp_3D_k_firstorder_10Percentile,

lbp_3D_k_firstorder_90Percentile,

lbp_3D_k_firstorder_Energy,

lbp_3D_k_firstorder_Entropy,

lbp_3D_k_firstorder_InterquartileRange,

lbp_3D_k_firstorder_Kurtosis,

lbp_3D_k_firstorder_Maximum,

lbp_3D_k_firstorder_Mean,

lbp_3D_k_firstorder_MeanAbsoluteDeviation,

lbp_3D_k_firstorder_Median,

lbp_3D_k_firstorder_Minimum,

lbp_3D_k_firstorder_Range,

lbp_3D_k_firstorder_RobustMeanAbsoluteDeviation,

lbp_3D_k_firstorder_RootMeanSquared,

lbp_3D_k_firstorder_Skewness,

lbp_3D_k_firstorder_TotalEnergy,

lbp_3D_k_firstorder_Uniformity,

lbp_3D_k_firstorder_Variance,

lbp_3D_k_glcm_Autocorrelation,

lbp_3D_k_glcm_ClusterProminence,

lbp_3D_k_glcm_ClusterShade,

lbp_3D_k_glcm_ClusterTendency,

lbp_3D_k_glcm_Contrast,

lbp_3D_k_glcm_Correlation,

lbp_3D_k_glcm_DifferenceAverage,

lbp_3D_k_glcm_DifferenceEntropy,

lbp_3D_k_glcm_DifferenceVariance,

lbp_3D_k_glcm_Id,

lbp_3D_k_glcm_Idm,

lbp_3D_k_glcm_Idmn,

lbp_3D_k_glcm_Idn,

lbp_3D_k_glcm_Imc1,

lbp_3D_k_glcm_Imc2,

lbp_3D_k_glcm_InverseVariance,

lbp_3D_k_glcm_JointAverage,

lbp_3D_k_glcm_JointEnergy,

lbp_3D_k_glcm_JointEntropy,

lbp_3D_k_glcm_MaximumProbability,

lbp_3D_k_glcm_SumEntropy,

lbp_3D_k_glcm_SumSquares,

lbp_3D_k_gldm_DependenceEntropy,

lbp_3D_k_gldm_DependenceNonUniformity,

lbp_3D_k_gldm_DependenceNonUniformityNormalized,

lbp_3D_k_gldm_DependenceVariance,

lbp_3D_k_gldm_GrayLevelNonUniformity,

lbp_3D_k_gldm_GrayLevelVariance,

lbp_3D_k_gldm_HighGrayLevelEmphasis,

lbp_3D_k_gldm_LargeDependenceEmphasis,

lbp_3D_k_gldm_LargeDependenceHighGrayLevelEmphasis,

lbp_3D_k_gldm_LargeDependenceLowGrayLevelEmphasis,

lbp_3D_k_gldm_LowGrayLevelEmphasis,

lbp_3D_k_gldm_SmallDependenceEmphasis,

lbp_3D_k_gldm_SmallDependenceHighGrayLevelEmphasis,

lbp_3D_k_gldm_SmallDependenceLowGrayLevelEmphasis,

lbp_3D_k_glrlm_GrayLevelNonUniformity,

lbp_3D_k_glrlm_GrayLevelNonUniformityNormalized,

lbp_3D_k_glrlm_GrayLevelVariance,

lbp_3D_k_glrlm_HighGrayLevelRunEmphasis,

lbp_3D_k_glrlm_LongRunEmphasis,

lbp_3D_k_glrlm_LongRunHighGrayLevelEmphasis,

lbp_3D_k_glrlm_LongRunLowGrayLevelEmphasis,

lbp_3D_k_glrlm_LowGrayLevelRunEmphasis,

lbp_3D_k_glrlm_RunEntropy,

lbp_3D_k_glrlm_RunLengthNonUniformity,

lbp_3D_k_glrlm_RunLengthNonUniformityNormalized,

lbp_3D_k_glrlm_RunPercentage,

lbp_3D_k_glrlm_RunVariance,

lbp_3D_k_glrlm_ShortRunEmphasis,

lbp_3D_k_glrlm_ShortRunHighGrayLevelEmphasis,

lbp_3D_k_glrlm_ShortRunLowGrayLevelEmphasis,

lbp_3D_k_glszm_GrayLevelNonUniformity,

lbp_3D_k_glszm_GrayLevelNonUniformityNormalized,

lbp_3D_k_glszm_GrayLevelVariance,

lbp_3D_k_glszm_HighGrayLevelZoneEmphasis,

lbp_3D_k_glszm_LargeAreaEmphasis,

lbp_3D_k_glszm_LargeAreaHighGrayLevelEmphasis,

lbp_3D_k_glszm_LargeAreaLowGrayLevelEmphasis,

lbp_3D_k_glszm_LowGrayLevelZoneEmphasis,

lbp_3D_k_glszm_SizeZoneNonUniformity,

lbp_3D_k_glszm_SizeZoneNonUniformityNormalized,

lbp_3D_k_glszm_SmallAreaEmphasis,

lbp_3D_k_glszm_SmallAreaHighGrayLevelEmphasis,

lbp_3D_k_glszm_SmallAreaLowGrayLevelEmphasis,

lbp_3D_k_glszm_ZoneEntropy,

lbp_3D_k_glszm_ZonePercentage,

lbp_3D_k_glszm_ZoneVariance,

lbp_3D_k_ngtdm_Busyness,

lbp_3D_k_ngtdm_Coarseness,

lbp_3D_k_ngtdm_Complexity,

lbp_3D_k_ngtdm_Contrast,

lbp_3D_k_ngtdm_Strength,

lbp_3D_m1_firstorder_10Percentile,

lbp_3D_m1_firstorder_90Percentile,

lbp_3D_m1_firstorder_Energy,

lbp_3D_m1_firstorder_Entropy,

lbp_3D_m1_firstorder_InterquartileRange,

lbp_3D_m1_firstorder_Kurtosis,

lbp_3D_m1_firstorder_Maximum,

lbp_3D_m1_firstorder_Mean,

lbp_3D_m1_firstorder_MeanAbsoluteDeviation,

lbp_3D_m1_firstorder_Median,

lbp_3D_m1_firstorder_Minimum,

lbp_3D_m1_firstorder_Range,

lbp_3D_m1_firstorder_RobustMeanAbsoluteDeviation,

lbp_3D_m1_firstorder_RootMeanSquared,

lbp_3D_m1_firstorder_Skewness,

lbp_3D_m1_firstorder_TotalEnergy,

lbp_3D_m1_firstorder_Uniformity,

lbp_3D_m1_firstorder_Variance,

lbp_3D_m1_glcm_Autocorrelation,

lbp_3D_m1_glcm_ClusterProminence,

lbp_3D_m1_glcm_ClusterShade,

lbp_3D_m1_glcm_ClusterTendency,

lbp_3D_m1_glcm_Contrast,

lbp_3D_m1_glcm_Correlation,

lbp_3D_m1_glcm_DifferenceAverage,

lbp_3D_m1_glcm_DifferenceEntropy,

lbp_3D_m1_glcm_DifferenceVariance,

lbp_3D_m1_glcm_Id,

lbp_3D_m1_glcm_Idm,

lbp_3D_m1_glcm_Idmn,

lbp_3D_m1_glcm_Idn,

lbp_3D_m1_glcm_Imc1,

lbp_3D_m1_glcm_Imc2,

lbp_3D_m1_glcm_InverseVariance,

lbp_3D_m1_glcm_JointAverage,

lbp_3D_m1_glcm_JointEnergy,

lbp_3D_m1_glcm_JointEntropy,

lbp_3D_m1_glcm_MaximumProbability,

lbp_3D_m1_glcm_SumEntropy,

lbp_3D_m1_glcm_SumSquares,

lbp_3D_m1_gldm_DependenceEntropy,

lbp_3D_m1_gldm_DependenceNonUniformity,

lbp_3D_m1_gldm_DependenceNonUniformityNormalized,

lbp_3D_m1_gldm_DependenceVariance,

lbp_3D_m1_gldm_GrayLevelNonUniformity,

lbp_3D_m1_gldm_GrayLevelVariance,

lbp_3D_m1_gldm_HighGrayLevelEmphasis,

lbp_3D_m1_gldm_LargeDependenceEmphasis,

lbp_3D_m1_gldm_LargeDependenceHighGrayLevelEmphasis,

lbp_3D_m1_gldm_LargeDependenceLowGrayLevelEmphasis,

lbp_3D_m1_gldm_LowGrayLevelEmphasis,

lbp_3D_m1_gldm_SmallDependenceEmphasis,

lbp_3D_m1_gldm_SmallDependenceHighGrayLevelEmphasis,

lbp_3D_m1_gldm_SmallDependenceLowGrayLevelEmphasis,

lbp_3D_m1_glrlm_GrayLevelNonUniformity,

lbp_3D_m1_glrlm_GrayLevelNonUniformityNormalized,

lbp_3D_m1_glrlm_GrayLevelVariance,

lbp_3D_m1_glrlm_HighGrayLevelRunEmphasis,

lbp_3D_m1_glrlm_LongRunEmphasis,

lbp_3D_m1_glrlm_LongRunHighGrayLevelEmphasis,

lbp_3D_m1_glrlm_LongRunLowGrayLevelEmphasis,

lbp_3D_m1_glrlm_LowGrayLevelRunEmphasis,

lbp_3D_m1_glrlm_RunEntropy,

lbp_3D_m1_glrlm_RunLengthNonUniformity,

lbp_3D_m1_glrlm_RunLengthNonUniformityNormalized,

lbp_3D_m1_glrlm_RunPercentage,

lbp_3D_m1_glrlm_RunVariance,

lbp_3D_m1_glrlm_ShortRunEmphasis,

lbp_3D_m1_glrlm_ShortRunHighGrayLevelEmphasis,

lbp_3D_m1_glrlm_ShortRunLowGrayLevelEmphasis,

lbp_3D_m1_glszm_GrayLevelNonUniformity,

lbp_3D_m1_glszm_GrayLevelNonUniformityNormalized,

lbp_3D_m1_glszm_GrayLevelVariance,

lbp_3D_m1_glszm_HighGrayLevelZoneEmphasis,

lbp_3D_m1_glszm_LargeAreaEmphasis,

lbp_3D_m1_glszm_LargeAreaHighGrayLevelEmphasis,

lbp_3D_m1_glszm_LargeAreaLowGrayLevelEmphasis,

lbp_3D_m1_glszm_LowGrayLevelZoneEmphasis,

lbp_3D_m1_glszm_SizeZoneNonUniformity,

lbp_3D_m1_glszm_SizeZoneNonUniformityNormalized,

lbp_3D_m1_glszm_SmallAreaEmphasis,

lbp_3D_m1_glszm_SmallAreaHighGrayLevelEmphasis,

lbp_3D_m1_glszm_SmallAreaLowGrayLevelEmphasis,

lbp_3D_m1_glszm_ZoneEntropy,

lbp_3D_m1_glszm_ZonePercentage,

lbp_3D_m1_glszm_ZoneVariance,

lbp_3D_m1_ngtdm_Busyness,

lbp_3D_m1_ngtdm_Coarseness,

lbp_3D_m1_ngtdm_Complexity,

lbp_3D_m1_ngtdm_Contrast,

lbp_3D_m1_ngtdm_Strength,

lbp_3D_m2_firstorder_10Percentile,

lbp_3D_m2_firstorder_90Percentile,

lbp_3D_m2_firstorder_Energy,

lbp_3D_m2_firstorder_Entropy,

lbp_3D_m2_firstorder_InterquartileRange,

lbp_3D_m2_firstorder_Kurtosis,

lbp_3D_m2_firstorder_Maximum,

lbp_3D_m2_firstorder_Mean,

lbp_3D_m2_firstorder_MeanAbsoluteDeviation,

lbp_3D_m2_firstorder_Median,

lbp_3D_m2_firstorder_Minimum,

lbp_3D_m2_firstorder_Range,

lbp_3D_m2_firstorder_RobustMeanAbsoluteDeviation,

lbp_3D_m2_firstorder_RootMeanSquared,

lbp_3D_m2_firstorder_Skewness,

lbp_3D_m2_firstorder_TotalEnergy,

lbp_3D_m2_firstorder_Uniformity,

lbp_3D_m2_firstorder_Variance,

lbp_3D_m2_glcm_Autocorrelation,

lbp_3D_m2_glcm_ClusterProminence,

lbp_3D_m2_glcm_ClusterShade,

lbp_3D_m2_glcm_ClusterTendency,

lbp_3D_m2_glcm_Contrast,

lbp_3D_m2_glcm_Correlation,

lbp_3D_m2_glcm_DifferenceAverage,

lbp_3D_m2_glcm_DifferenceEntropy,

lbp_3D_m2_glcm_DifferenceVariance,

lbp_3D_m2_glcm_Id,

lbp_3D_m2_glcm_Idm,

lbp_3D_m2_glcm_Idmn,

lbp_3D_m2_glcm_Idn,

lbp_3D_m2_glcm_Imc1,

lbp_3D_m2_glcm_Imc2,

lbp_3D_m2_glcm_InverseVariance,

lbp_3D_m2_glcm_JointAverage,

lbp_3D_m2_glcm_JointEnergy,

lbp_3D_m2_glcm_JointEntropy,

lbp_3D_m2_glcm_MaximumProbability,

lbp_3D_m2_glcm_SumEntropy,

lbp_3D_m2_glcm_SumSquares,

lbp_3D_m2_gldm_DependenceEntropy,

lbp_3D_m2_gldm_DependenceNonUniformity,

lbp_3D_m2_gldm_DependenceNonUniformityNormalized,

lbp_3D_m2_gldm_DependenceVariance,

lbp_3D_m2_gldm_GrayLevelNonUniformity,

lbp_3D_m2_gldm_GrayLevelVariance,

lbp_3D_m2_gldm_HighGrayLevelEmphasis,

lbp_3D_m2_gldm_LargeDependenceEmphasis,

lbp_3D_m2_gldm_LargeDependenceHighGrayLevelEmphasis,

lbp_3D_m2_gldm_LargeDependenceLowGrayLevelEmphasis,

lbp_3D_m2_gldm_LowGrayLevelEmphasis,

lbp_3D_m2_gldm_SmallDependenceEmphasis,

lbp_3D_m2_gldm_SmallDependenceHighGrayLevelEmphasis,

lbp_3D_m2_gldm_SmallDependenceLowGrayLevelEmphasis,

lbp_3D_m2_glrlm_GrayLevelNonUniformity,

lbp_3D_m2_glrlm_GrayLevelNonUniformityNormalized,

lbp_3D_m2_glrlm_GrayLevelVariance,

lbp_3D_m2_glrlm_HighGrayLevelRunEmphasis,

lbp_3D_m2_glrlm_LongRunEmphasis,

lbp_3D_m2_glrlm_LongRunHighGrayLevelEmphasis,

lbp_3D_m2_glrlm_LongRunLowGrayLevelEmphasis,

lbp_3D_m2_glrlm_LowGrayLevelRunEmphasis,

lbp_3D_m2_glrlm_RunEntropy,

lbp_3D_m2_glrlm_RunLengthNonUniformity,

lbp_3D_m2_glrlm_RunLengthNonUniformityNormalized,

lbp_3D_m2_glrlm_RunPercentage,

lbp_3D_m2_glrlm_RunVariance,

lbp_3D_m2_glrlm_ShortRunEmphasis,

lbp_3D_m2_glrlm_ShortRunHighGrayLevelEmphasis,

lbp_3D_m2_glrlm_ShortRunLowGrayLevelEmphasis,

lbp_3D_m2_glszm_GrayLevelNonUniformity,

lbp_3D_m2_glszm_GrayLevelNonUniformityNormalized,

lbp_3D_m2_glszm_GrayLevelVariance,

lbp_3D_m2_glszm_HighGrayLevelZoneEmphasis,

lbp_3D_m2_glszm_LargeAreaEmphasis,

lbp_3D_m2_glszm_LargeAreaHighGrayLevelEmphasis,

lbp_3D_m2_glszm_LargeAreaLowGrayLevelEmphasis,

lbp_3D_m2_glszm_LowGrayLevelZoneEmphasis,

lbp_3D_m2_glszm_SizeZoneNonUniformity,

lbp_3D_m2_glszm_SizeZoneNonUniformityNormalized,

lbp_3D_m2_glszm_SmallAreaEmphasis,

lbp_3D_m2_glszm_SmallAreaHighGrayLevelEmphasis,

lbp_3D_m2_glszm_SmallAreaLowGrayLevelEmphasis,

lbp_3D_m2_glszm_ZoneEntropy,

lbp_3D_m2_glszm_ZonePercentage,

lbp_3D_m2_glszm_ZoneVariance,

lbp_3D_m2_ngtdm_Busyness,

lbp_3D_m2_ngtdm_Coarseness,

lbp_3D_m2_ngtdm_Complexity,

lbp_3D_m2_ngtdm_Contrast,

lbp_3D_m2_ngtdm_Strength,

log_sigma_1_0_mm_3D_firstorder_10Percentile,

log_sigma_1_0_mm_3D_firstorder_90Percentile,

log_sigma_1_0_mm_3D_firstorder_Energy,

log_sigma_1_0_mm_3D_firstorder_Entropy,

log_sigma_1_0_mm_3D_firstorder_InterquartileRange,

log_sigma_1_0_mm_3D_firstorder_Kurtosis,

log_sigma_1_0_mm_3D_firstorder_Maximum,

log_sigma_1_0_mm_3D_firstorder_Mean,

log_sigma_1_0_mm_3D_firstorder_MeanAbsoluteDeviation,

log_sigma_1_0_mm_3D_firstorder_Median,

log_sigma_1_0_mm_3D_firstorder_Minimum,

log_sigma_1_0_mm_3D_firstorder_Range,

log_sigma_1_0_mm_3D_firstorder_RobustMeanAbsoluteDeviation,

log_sigma_1_0_mm_3D_firstorder_RootMeanSquared,

log_sigma_1_0_mm_3D_firstorder_Skewness,

log_sigma_1_0_mm_3D_firstorder_TotalEnergy,

log_sigma_1_0_mm_3D_firstorder_Uniformity,

log_sigma_1_0_mm_3D_firstorder_Variance,

log_sigma_1_0_mm_3D_glcm_Autocorrelation,

log_sigma_1_0_mm_3D_glcm_ClusterProminence,

log_sigma_1_0_mm_3D_glcm_ClusterShade,

log_sigma_1_0_mm_3D_glcm_ClusterTendency,

log_sigma_1_0_mm_3D_glcm_Contrast,

log_sigma_1_0_mm_3D_glcm_Correlation,

log_sigma_1_0_mm_3D_glcm_DifferenceAverage,

log_sigma_1_0_mm_3D_glcm_DifferenceEntropy,

log_sigma_1_0_mm_3D_glcm_DifferenceVariance,

log_sigma_1_0_mm_3D_glcm_Id,

log_sigma_1_0_mm_3D_glcm_Idm,

log_sigma_1_0_mm_3D_glcm_Idmn,

log_sigma_1_0_mm_3D_glcm_Idn,

log_sigma_1_0_mm_3D_glcm_Imc1,

log_sigma_1_0_mm_3D_glcm_Imc2,

log_sigma_1_0_mm_3D_glcm_InverseVariance,

log_sigma_1_0_mm_3D_glcm_JointAverage,

log_sigma_1_0_mm_3D_glcm_JointEnergy,

log_sigma_1_0_mm_3D_glcm_JointEntropy,

log_sigma_1_0_mm_3D_glcm_MaximumProbability,

log_sigma_1_0_mm_3D_glcm_SumEntropy,

log_sigma_1_0_mm_3D_glcm_SumSquares,

log_sigma_1_0_mm_3D_gldm_DependenceEntropy,

log_sigma_1_0_mm_3D_gldm_DependenceNonUniformity,

log_sigma_1_0_mm_3D_gldm_DependenceNonUniformityNormalized,

log_sigma_1_0_mm_3D_gldm_DependenceVariance,

log_sigma_1_0_mm_3D_gldm_GrayLevelNonUniformity,

log_sigma_1_0_mm_3D_gldm_GrayLevelVariance,

log_sigma_1_0_mm_3D_gldm_HighGrayLevelEmphasis,

log_sigma_1_0_mm_3D_gldm_LargeDependenceEmphasis,

log_sigma_1_0_mm_3D_gldm_LargeDependenceHighGrayLevelEmphasis,

log_sigma_1_0_mm_3D_gldm_LargeDependenceLowGrayLevelEmphasis,

log_sigma_1_0_mm_3D_gldm_LowGrayLevelEmphasis,

log_sigma_1_0_mm_3D_gldm_SmallDependenceEmphasis,

log_sigma_1_0_mm_3D_gldm_SmallDependenceHighGrayLevelEmphasis,

log_sigma_1_0_mm_3D_gldm_SmallDependenceLowGrayLevelEmphasis,

log_sigma_1_0_mm_3D_glrlm_GrayLevelNonUniformity,

log_sigma_1_0_mm_3D_glrlm_GrayLevelNonUniformityNormalized,

log_sigma_1_0_mm_3D_glrlm_GrayLevelVariance,

log_sigma_1_0_mm_3D_glrlm_HighGrayLevelRunEmphasis,

log_sigma_1_0_mm_3D_glrlm_LongRunEmphasis,

log_sigma_1_0_mm_3D_glrlm_LongRunHighGrayLevelEmphasis,

log_sigma_1_0_mm_3D_glrlm_LongRunLowGrayLevelEmphasis,

log_sigma_1_0_mm_3D_glrlm_LowGrayLevelRunEmphasis,

log_sigma_1_0_mm_3D_glrlm_RunEntropy,

log_sigma_1_0_mm_3D_glrlm_RunLengthNonUniformity,

log_sigma_1_0_mm_3D_glrlm_RunLengthNonUniformityNormalized,

log_sigma_1_0_mm_3D_glrlm_RunPercentage,

log_sigma_1_0_mm_3D_glrlm_RunVariance,

log_sigma_1_0_mm_3D_glrlm_ShortRunEmphasis,

log_sigma_1_0_mm_3D_glrlm_ShortRunHighGrayLevelEmphasis,

log_sigma_1_0_mm_3D_glrlm_ShortRunLowGrayLevelEmphasis,

log_sigma_1_0_mm_3D_glszm_GrayLevelNonUniformity,

log_sigma_1_0_mm_3D_glszm_GrayLevelNonUniformityNormalized,

log_sigma_1_0_mm_3D_glszm_GrayLevelVariance,

log_sigma_1_0_mm_3D_glszm_HighGrayLevelZoneEmphasis,

log_sigma_1_0_mm_3D_glszm_LargeAreaEmphasis,

log_sigma_1_0_mm_3D_glszm_LargeAreaHighGrayLevelEmphasis,

log_sigma_1_0_mm_3D_glszm_LargeAreaLowGrayLevelEmphasis,

log_sigma_1_0_mm_3D_glszm_LowGrayLevelZoneEmphasis,

log_sigma_1_0_mm_3D_glszm_SizeZoneNonUniformity,

log_sigma_1_0_mm_3D_glszm_SizeZoneNonUniformityNormalized,

log_sigma_1_0_mm_3D_glszm_SmallAreaEmphasis,

log_sigma_1_0_mm_3D_glszm_SmallAreaHighGrayLevelEmphasis,

log_sigma_1_0_mm_3D_glszm_SmallAreaLowGrayLevelEmphasis,

log_sigma_1_0_mm_3D_glszm_ZoneEntropy,

log_sigma_1_0_mm_3D_glszm_ZonePercentage,

log_sigma_1_0_mm_3D_glszm_ZoneVariance,

log_sigma_1_0_mm_3D_ngtdm_Busyness,

log_sigma_1_0_mm_3D_ngtdm_Coarseness,

log_sigma_1_0_mm_3D_ngtdm_Complexity,

log_sigma_1_0_mm_3D_ngtdm_Contrast,

log_sigma_1_0_mm_3D_ngtdm_Strength,

log_sigma_2_0_mm_3D_firstorder_10Percentile,

log_sigma_2_0_mm_3D_firstorder_90Percentile,

log_sigma_2_0_mm_3D_firstorder_Energy,

log_sigma_2_0_mm_3D_firstorder_Entropy,

log_sigma_2_0_mm_3D_firstorder_InterquartileRange,

log_sigma_2_0_mm_3D_firstorder_Kurtosis,

log_sigma_2_0_mm_3D_firstorder_Maximum,

log_sigma_2_0_mm_3D_firstorder_Mean,

log_sigma_2_0_mm_3D_firstorder_MeanAbsoluteDeviation,

log_sigma_2_0_mm_3D_firstorder_Median,

log_sigma_2_0_mm_3D_firstorder_Minimum,

log_sigma_2_0_mm_3D_firstorder_Range,

log_sigma_2_0_mm_3D_firstorder_RobustMeanAbsoluteDeviation,

log_sigma_2_0_mm_3D_firstorder_RootMeanSquared,

log_sigma_2_0_mm_3D_firstorder_Skewness,

log_sigma_2_0_mm_3D_firstorder_TotalEnergy,

log_sigma_2_0_mm_3D_firstorder_Uniformity,

log_sigma_2_0_mm_3D_firstorder_Variance,

log_sigma_2_0_mm_3D_glcm_Autocorrelation,

log_sigma_2_0_mm_3D_glcm_ClusterProminence,

log_sigma_2_0_mm_3D_glcm_ClusterShade,

log_sigma_2_0_mm_3D_glcm_ClusterTendency,

log_sigma_2_0_mm_3D_glcm_Contrast,

log_sigma_2_0_mm_3D_glcm_Correlation,

log_sigma_2_0_mm_3D_glcm_DifferenceAverage,

log_sigma_2_0_mm_3D_glcm_DifferenceEntropy,

log_sigma_2_0_mm_3D_glcm_DifferenceVariance,

log_sigma_2_0_mm_3D_glcm_Id,

log_sigma_2_0_mm_3D_glcm_Idm,

log_sigma_2_0_mm_3D_glcm_Idmn,

log_sigma_2_0_mm_3D_glcm_Idn,

log_sigma_2_0_mm_3D_glcm_Imc1,

log_sigma_2_0_mm_3D_glcm_Imc2,

log_sigma_2_0_mm_3D_glcm_InverseVariance,

log_sigma_2_0_mm_3D_glcm_JointAverage,

log_sigma_2_0_mm_3D_glcm_JointEnergy,

log_sigma_2_0_mm_3D_glcm_JointEntropy,

log_sigma_2_0_mm_3D_glcm_MaximumProbability,

log_sigma_2_0_mm_3D_glcm_SumEntropy,

log_sigma_2_0_mm_3D_glcm_SumSquares,

log_sigma_2_0_mm_3D_gldm_DependenceEntropy,

log_sigma_2_0_mm_3D_gldm_DependenceNonUniformity,

log_sigma_2_0_mm_3D_gldm_DependenceNonUniformityNormalized,

log_sigma_2_0_mm_3D_gldm_DependenceVariance,

log_sigma_2_0_mm_3D_gldm_GrayLevelNonUniformity,

log_sigma_2_0_mm_3D_gldm_GrayLevelVariance,

log_sigma_2_0_mm_3D_gldm_HighGrayLevelEmphasis,

log_sigma_2_0_mm_3D_gldm_LargeDependenceEmphasis,

log_sigma_2_0_mm_3D_gldm_LargeDependenceHighGrayLevelEmphasis,

log_sigma_2_0_mm_3D_gldm_LargeDependenceLowGrayLevelEmphasis,

log_sigma_2_0_mm_3D_gldm_LowGrayLevelEmphasis,

log_sigma_2_0_mm_3D_gldm_SmallDependenceEmphasis,

log_sigma_2_0_mm_3D_gldm_SmallDependenceHighGrayLevelEmphasis,

log_sigma_2_0_mm_3D_gldm_SmallDependenceLowGrayLevelEmphasis,

log_sigma_2_0_mm_3D_glrlm_GrayLevelNonUniformity,

log_sigma_2_0_mm_3D_glrlm_GrayLevelNonUniformityNormalized,

log_sigma_2_0_mm_3D_glrlm_GrayLevelVariance,

log_sigma_2_0_mm_3D_glrlm_HighGrayLevelRunEmphasis,

log_sigma_2_0_mm_3D_glrlm_LongRunEmphasis,

log_sigma_2_0_mm_3D_glrlm_LongRunHighGrayLevelEmphasis,

log_sigma_2_0_mm_3D_glrlm_LongRunLowGrayLevelEmphasis,

log_sigma_2_0_mm_3D_glrlm_LowGrayLevelRunEmphasis,

log_sigma_2_0_mm_3D_glrlm_RunEntropy,

log_sigma_2_0_mm_3D_glrlm_RunLengthNonUniformity,

log_sigma_2_0_mm_3D_glrlm_RunLengthNonUniformityNormalized,

log_sigma_2_0_mm_3D_glrlm_RunPercentage,

log_sigma_2_0_mm_3D_glrlm_RunVariance,

log_sigma_2_0_mm_3D_glrlm_ShortRunEmphasis,

log_sigma_2_0_mm_3D_glrlm_ShortRunHighGrayLevelEmphasis,

log_sigma_2_0_mm_3D_glrlm_ShortRunLowGrayLevelEmphasis,

log_sigma_2_0_mm_3D_glszm_GrayLevelNonUniformity,

log_sigma_2_0_mm_3D_glszm_GrayLevelNonUniformityNormalized,

log_sigma_2_0_mm_3D_glszm_GrayLevelVariance,

log_sigma_2_0_mm_3D_glszm_HighGrayLevelZoneEmphasis,

log_sigma_2_0_mm_3D_glszm_LargeAreaEmphasis,

log_sigma_2_0_mm_3D_glszm_LargeAreaHighGrayLevelEmphasis,

log_sigma_2_0_mm_3D_glszm_LargeAreaLowGrayLevelEmphasis,

log_sigma_2_0_mm_3D_glszm_LowGrayLevelZoneEmphasis,

log_sigma_2_0_mm_3D_glszm_SizeZoneNonUniformity,

log_sigma_2_0_mm_3D_glszm_SizeZoneNonUniformityNormalized,

log_sigma_2_0_mm_3D_glszm_SmallAreaEmphasis,

log_sigma_2_0_mm_3D_glszm_SmallAreaHighGrayLevelEmphasis,

log_sigma_2_0_mm_3D_glszm_SmallAreaLowGrayLevelEmphasis,

log_sigma_2_0_mm_3D_glszm_ZoneEntropy,

log_sigma_2_0_mm_3D_glszm_ZonePercentage,

log_sigma_2_0_mm_3D_glszm_ZoneVariance,

log_sigma_2_0_mm_3D_ngtdm_Busyness,

log_sigma_2_0_mm_3D_ngtdm_Coarseness,

log_sigma_2_0_mm_3D_ngtdm_Complexity,

log_sigma_2_0_mm_3D_ngtdm_Contrast,

log_sigma_2_0_mm_3D_ngtdm_Strength,

log_sigma_3_0_mm_3D_firstorder_10Percentile,

log_sigma_3_0_mm_3D_firstorder_90Percentile,

log_sigma_3_0_mm_3D_firstorder_Energy,

log_sigma_3_0_mm_3D_firstorder_Entropy,

log_sigma_3_0_mm_3D_firstorder_InterquartileRange,

log_sigma_3_0_mm_3D_firstorder_Kurtosis,

log_sigma_3_0_mm_3D_firstorder_Maximum,

log_sigma_3_0_mm_3D_firstorder_Mean,

log_sigma_3_0_mm_3D_firstorder_MeanAbsoluteDeviation,

log_sigma_3_0_mm_3D_firstorder_Median,

log_sigma_3_0_mm_3D_firstorder_Minimum,

log_sigma_3_0_mm_3D_firstorder_Range,

log_sigma_3_0_mm_3D_firstorder_RobustMeanAbsoluteDeviation,

log_sigma_3_0_mm_3D_firstorder_RootMeanSquared,

log_sigma_3_0_mm_3D_firstorder_Skewness,

log_sigma_3_0_mm_3D_firstorder_TotalEnergy,

log_sigma_3_0_mm_3D_firstorder_Uniformity,

log_sigma_3_0_mm_3D_firstorder_Variance,

log_sigma_3_0_mm_3D_glcm_Autocorrelation,

log_sigma_3_0_mm_3D_glcm_ClusterProminence,

log_sigma_3_0_mm_3D_glcm_ClusterShade,

log_sigma_3_0_mm_3D_glcm_ClusterTendency,

log_sigma_3_0_mm_3D_glcm_Contrast,

log_sigma_3_0_mm_3D_glcm_Correlation,

log_sigma_3_0_mm_3D_glcm_DifferenceAverage,

log_sigma_3_0_mm_3D_glcm_DifferenceEntropy,

log_sigma_3_0_mm_3D_glcm_DifferenceVariance,

log_sigma_3_0_mm_3D_glcm_Id,

log_sigma_3_0_mm_3D_glcm_Idm,

log_sigma_3_0_mm_3D_glcm_Idmn,

log_sigma_3_0_mm_3D_glcm_Idn,

log_sigma_3_0_mm_3D_glcm_Imc1,

log_sigma_3_0_mm_3D_glcm_Imc2,

log_sigma_3_0_mm_3D_glcm_InverseVariance,

log_sigma_3_0_mm_3D_glcm_JointAverage,

log_sigma_3_0_mm_3D_glcm_JointEnergy,

log_sigma_3_0_mm_3D_glcm_JointEntropy,

log_sigma_3_0_mm_3D_glcm_MaximumProbability,

log_sigma_3_0_mm_3D_glcm_SumEntropy,

log_sigma_3_0_mm_3D_glcm_SumSquares,

log_sigma_3_0_mm_3D_gldm_DependenceEntropy,

log_sigma_3_0_mm_3D_gldm_DependenceNonUniformity,

log_sigma_3_0_mm_3D_gldm_DependenceNonUniformityNormalized,

log_sigma_3_0_mm_3D_gldm_DependenceVariance,

log_sigma_3_0_mm_3D_gldm_GrayLevelNonUniformity,

log_sigma_3_0_mm_3D_gldm_GrayLevelVariance,

log_sigma_3_0_mm_3D_gldm_HighGrayLevelEmphasis,

log_sigma_3_0_mm_3D_gldm_LargeDependenceEmphasis,

log_sigma_3_0_mm_3D_gldm_LargeDependenceHighGrayLevelEmphasis,

log_sigma_3_0_mm_3D_gldm_LargeDependenceLowGrayLevelEmphasis,

log_sigma_3_0_mm_3D_gldm_LowGrayLevelEmphasis,

log_sigma_3_0_mm_3D_gldm_SmallDependenceEmphasis,

log_sigma_3_0_mm_3D_gldm_SmallDependenceHighGrayLevelEmphasis,

log_sigma_3_0_mm_3D_gldm_SmallDependenceLowGrayLevelEmphasis,

log_sigma_3_0_mm_3D_glrlm_GrayLevelNonUniformity,

log_sigma_3_0_mm_3D_glrlm_GrayLevelNonUniformityNormalized,

log_sigma_3_0_mm_3D_glrlm_GrayLevelVariance,

log_sigma_3_0_mm_3D_glrlm_HighGrayLevelRunEmphasis,

log_sigma_3_0_mm_3D_glrlm_LongRunEmphasis,

log_sigma_3_0_mm_3D_glrlm_LongRunHighGrayLevelEmphasis,

log_sigma_3_0_mm_3D_glrlm_LongRunLowGrayLevelEmphasis,

log_sigma_3_0_mm_3D_glrlm_LowGrayLevelRunEmphasis,

log_sigma_3_0_mm_3D_glrlm_RunEntropy,

log_sigma_3_0_mm_3D_glrlm_RunLengthNonUniformity,

log_sigma_3_0_mm_3D_glrlm_RunLengthNonUniformityNormalized,

log_sigma_3_0_mm_3D_glrlm_RunPercentage,

log_sigma_3_0_mm_3D_glrlm_RunVariance,

log_sigma_3_0_mm_3D_glrlm_ShortRunEmphasis,

log_sigma_3_0_mm_3D_glrlm_ShortRunHighGrayLevelEmphasis,

log_sigma_3_0_mm_3D_glrlm_ShortRunLowGrayLevelEmphasis,

log_sigma_3_0_mm_3D_glszm_GrayLevelNonUniformity,

log_sigma_3_0_mm_3D_glszm_GrayLevelNonUniformityNormalized,

log_sigma_3_0_mm_3D_glszm_GrayLevelVariance,

log_sigma_3_0_mm_3D_glszm_HighGrayLevelZoneEmphasis,

log_sigma_3_0_mm_3D_glszm_LargeAreaEmphasis,

log_sigma_3_0_mm_3D_glszm_LargeAreaHighGrayLevelEmphasis,

log_sigma_3_0_mm_3D_glszm_LargeAreaLowGrayLevelEmphasis,

log_sigma_3_0_mm_3D_glszm_LowGrayLevelZoneEmphasis,

log_sigma_3_0_mm_3D_glszm_SizeZoneNonUniformity,

log_sigma_3_0_mm_3D_glszm_SizeZoneNonUniformityNormalized,

log_sigma_3_0_mm_3D_glszm_SmallAreaEmphasis,

log_sigma_3_0_mm_3D_glszm_SmallAreaHighGrayLevelEmphasis,

log_sigma_3_0_mm_3D_glszm_SmallAreaLowGrayLevelEmphasis,

log_sigma_3_0_mm_3D_glszm_ZoneEntropy,

log_sigma_3_0_mm_3D_glszm_ZonePercentage,

log_sigma_3_0_mm_3D_glszm_ZoneVariance,

log_sigma_3_0_mm_3D_ngtdm_Busyness,

log_sigma_3_0_mm_3D_ngtdm_Coarseness,

log_sigma_3_0_mm_3D_ngtdm_Complexity,

log_sigma_3_0_mm_3D_ngtdm_Contrast,

log_sigma_3_0_mm_3D_ngtdm_Strength,

logarithm_firstorder_10Percentile,

logarithm_firstorder_90Percentile,

logarithm_firstorder_Energy,

logarithm_firstorder_Entropy,

logarithm_firstorder_InterquartileRange,

logarithm_firstorder_Kurtosis,

logarithm_firstorder_Maximum,

logarithm_firstorder_Mean,

logarithm_firstorder_MeanAbsoluteDeviation,

logarithm_firstorder_Median,

logarithm_firstorder_Minimum,

logarithm_firstorder_Range,

logarithm_firstorder_RobustMeanAbsoluteDeviation,

logarithm_firstorder_RootMeanSquared,

logarithm_firstorder_Skewness,

logarithm_firstorder_TotalEnergy,

logarithm_firstorder_Uniformity,

logarithm_firstorder_Variance,

logarithm_glcm_Autocorrelation,

logarithm_glcm_ClusterProminence,

logarithm_glcm_ClusterShade,

logarithm_glcm_ClusterTendency,

logarithm_glcm_Contrast,

logarithm_glcm_Correlation,

logarithm_glcm_DifferenceAverage,

logarithm_glcm_DifferenceEntropy,

logarithm_glcm_DifferenceVariance,

logarithm_glcm_Id,

logarithm_glcm_Idm,

logarithm_glcm_Idmn,

logarithm_glcm_Idn,

logarithm_glcm_Imc1,

logarithm_glcm_Imc2,

logarithm_glcm_InverseVariance,

logarithm_glcm_JointAverage,

logarithm_glcm_JointEnergy,

logarithm_glcm_JointEntropy,

logarithm_glcm_MaximumProbability,

logarithm_glcm_SumEntropy,

logarithm_glcm_SumSquares,

logarithm_gldm_DependenceEntropy,

logarithm_gldm_DependenceNonUniformity,

logarithm_gldm_DependenceNonUniformityNormalized,

logarithm_gldm_DependenceVariance,

logarithm_gldm_GrayLevelNonUniformity,

logarithm_gldm_GrayLevelVariance,

logarithm_gldm_HighGrayLevelEmphasis,

logarithm_gldm_LargeDependenceEmphasis,

logarithm_gldm_LargeDependenceHighGrayLevelEmphasis,

logarithm_gldm_LargeDependenceLowGrayLevelEmphasis,

logarithm_gldm_LowGrayLevelEmphasis,

logarithm_gldm_SmallDependenceEmphasis,

logarithm_gldm_SmallDependenceHighGrayLevelEmphasis,

logarithm_gldm_SmallDependenceLowGrayLevelEmphasis,

logarithm_glrlm_GrayLevelNonUniformity,

logarithm_glrlm_GrayLevelNonUniformityNormalized,

logarithm_glrlm_GrayLevelVariance,

logarithm_glrlm_HighGrayLevelRunEmphasis,

logarithm_glrlm_LongRunEmphasis,

logarithm_glrlm_LongRunHighGrayLevelEmphasis,

logarithm_glrlm_LongRunLowGrayLevelEmphasis,

logarithm_glrlm_LowGrayLevelRunEmphasis,

logarithm_glrlm_RunEntropy,

logarithm_glrlm_RunLengthNonUniformity,

logarithm_glrlm_RunLengthNonUniformityNormalized,

logarithm_glrlm_RunPercentage,

logarithm_glrlm_RunVariance,

logarithm_glrlm_ShortRunEmphasis,

logarithm_glrlm_ShortRunHighGrayLevelEmphasis,

logarithm_glrlm_ShortRunLowGrayLevelEmphasis,

logarithm_glszm_GrayLevelNonUniformity,

logarithm_glszm_GrayLevelNonUniformityNormalized,

logarithm_glszm_GrayLevelVariance,

logarithm_glszm_HighGrayLevelZoneEmphasis,

logarithm_glszm_LargeAreaEmphasis,

logarithm_glszm_LargeAreaHighGrayLevelEmphasis,

logarithm_glszm_LargeAreaLowGrayLevelEmphasis,

logarithm_glszm_LowGrayLevelZoneEmphasis,

logarithm_glszm_SizeZoneNonUniformity,

logarithm_glszm_SizeZoneNonUniformityNormalized,

logarithm_glszm_SmallAreaEmphasis,

logarithm_glszm_SmallAreaHighGrayLevelEmphasis,

logarithm_glszm_SmallAreaLowGrayLevelEmphasis,

logarithm_glszm_ZoneEntropy,

logarithm_glszm_ZonePercentage,

logarithm_glszm_ZoneVariance,

logarithm_ngtdm_Busyness,

logarithm_ngtdm_Coarseness,

logarithm_ngtdm_Complexity,

logarithm_ngtdm_Contrast,

logarithm_ngtdm_Strength,

original_firstorder_10Percentile,

original_firstorder_90Percentile,

original_firstorder_Energy,

original_firstorder_Entropy,

original_firstorder_InterquartileRange,

original_firstorder_Kurtosis,

original_firstorder_Maximum,

original_firstorder_Mean,

original_firstorder_MeanAbsoluteDeviation,

original_firstorder_Median,

original_firstorder_Minimum,

original_firstorder_Range,

original_firstorder_RobustMeanAbsoluteDeviation,

original_firstorder_RootMeanSquared,

original_firstorder_Skewness,

original_firstorder_TotalEnergy,

original_firstorder_Uniformity,

original_firstorder_Variance,

original_glcm_Autocorrelation,

original_glcm_ClusterProminence,

original_glcm_ClusterShade,

original_glcm_ClusterTendency,

original_glcm_Contrast,

original_glcm_Correlation,

original_glcm_DifferenceAverage,

original_glcm_DifferenceEntropy,

original_glcm_DifferenceVariance,

original_glcm_Id,

original_glcm_Idm,

original_glcm_Idmn,

original_glcm_Idn,

original_glcm_Imc1,

original_glcm_Imc2,

original_glcm_InverseVariance,

original_glcm_JointAverage,

original_glcm_JointEnergy,

original_glcm_JointEntropy,

original_glcm_MaximumProbability,

original_glcm_SumEntropy,

original_glcm_SumSquares,

original_gldm_DependenceEntropy,

original_gldm_DependenceNonUniformity,

original_gldm_DependenceNonUniformityNormalized,

original_gldm_DependenceVariance,

original_gldm_GrayLevelNonUniformity,

original_gldm_GrayLevelVariance,

original_gldm_HighGrayLevelEmphasis,

original_gldm_LargeDependenceEmphasis,

original_gldm_LargeDependenceHighGrayLevelEmphasis,

original_gldm_LargeDependenceLowGrayLevelEmphasis,

original_gldm_LowGrayLevelEmphasis,

original_gldm_SmallDependenceEmphasis,

original_gldm_SmallDependenceHighGrayLevelEmphasis,

original_gldm_SmallDependenceLowGrayLevelEmphasis,

original_glrlm_GrayLevelNonUniformity,

original_glrlm_GrayLevelNonUniformityNormalized,

original_glrlm_GrayLevelVariance,

original_glrlm_HighGrayLevelRunEmphasis,

original_glrlm_LongRunEmphasis,

original_glrlm_LongRunHighGrayLevelEmphasis,

original_glrlm_LongRunLowGrayLevelEmphasis,

original_glrlm_LowGrayLevelRunEmphasis,

original_glrlm_RunEntropy,

original_glrlm_RunLengthNonUniformity,

original_glrlm_RunLengthNonUniformityNormalized,

original_glrlm_RunPercentage,

original_glrlm_RunVariance,

original_glrlm_ShortRunEmphasis,

original_glrlm_ShortRunHighGrayLevelEmphasis,

original_glrlm_ShortRunLowGrayLevelEmphasis,

original_glszm_GrayLevelNonUniformity,

original_glszm_GrayLevelNonUniformityNormalized,

original_glszm_GrayLevelVariance,

original_glszm_HighGrayLevelZoneEmphasis,

original_glszm_LargeAreaEmphasis,

original_glszm_LargeAreaHighGrayLevelEmphasis,

original_glszm_LargeAreaLowGrayLevelEmphasis,

original_glszm_LowGrayLevelZoneEmphasis,

original_glszm_SizeZoneNonUniformity,

original_glszm_SizeZoneNonUniformityNormalized,

original_glszm_SmallAreaEmphasis,

original_glszm_SmallAreaHighGrayLevelEmphasis,

original_glszm_SmallAreaLowGrayLevelEmphasis,

original_glszm_ZoneEntropy,

original_glszm_ZonePercentage,

original_glszm_ZoneVariance,

original_ngtdm_Busyness,

original_ngtdm_Coarseness,

original_ngtdm_Complexity,

original_ngtdm_Contrast,

original_ngtdm_Strength,

original_shape_Elongation,

original_shape_Flatness,

original_shape_LeastAxisLength,

original_shape_MajorAxisLength,

original_shape_Maximum2DDiameterColumn,

original_shape_Maximum2DDiameterRow,

original_shape_Maximum2DDiameterSlice,

original_shape_Maximum3DDiameter,

original_shape_MeshVolume,

original_shape_MinorAxisLength,

original_shape_Sphericity,

original_shape_SurfaceArea,

original_shape_SurfaceVolumeRatio,

original_shape_VoxelVolume,

square_firstorder_10Percentile,

square_firstorder_90Percentile,

square_firstorder_Energy,

square_firstorder_Entropy,

square_firstorder_InterquartileRange,

square_firstorder_Kurtosis,

square_firstorder_Maximum,

square_firstorder_Mean,

square_firstorder_MeanAbsoluteDeviation,

square_firstorder_Median,

square_firstorder_Minimum,

square_firstorder_Range,

square_firstorder_RobustMeanAbsoluteDeviation,

square_firstorder_RootMeanSquared,

square_firstorder_Skewness,

square_firstorder_TotalEnergy,

square_firstorder_Uniformity,

square_firstorder_Variance,

square_glcm_Autocorrelation,

square_glcm_ClusterProminence,

square_glcm_ClusterShade,

square_glcm_ClusterTendency,

square_glcm_Contrast,

square_glcm_Correlation,

square_glcm_DifferenceAverage,

square_glcm_DifferenceEntropy,

square_glcm_DifferenceVariance,

square_glcm_Id,

square_glcm_Idm,

square_glcm_Idmn,

square_glcm_Idn,

square_glcm_Imc1,

square_glcm_Imc2,

square_glcm_InverseVariance,

square_glcm_JointAverage,

square_glcm_JointEnergy,

square_glcm_JointEntropy,

square_glcm_MaximumProbability,

square_glcm_SumEntropy,

square_glcm_SumSquares,

square_gldm_DependenceEntropy,

square_gldm_DependenceNonUniformity,

square_gldm_DependenceNonUniformityNormalized,

square_gldm_DependenceVariance,

square_gldm_GrayLevelNonUniformity,

square_gldm_GrayLevelVariance,

square_gldm_HighGrayLevelEmphasis,

square_gldm_LargeDependenceEmphasis,

square_gldm_LargeDependenceHighGrayLevelEmphasis,

square_gldm_LargeDependenceLowGrayLevelEmphasis,

square_gldm_LowGrayLevelEmphasis,

square_gldm_SmallDependenceEmphasis,

square_gldm_SmallDependenceHighGrayLevelEmphasis,

square_gldm_SmallDependenceLowGrayLevelEmphasis,

square_glrlm_GrayLevelNonUniformity,

square_glrlm_GrayLevelNonUniformityNormalized,

square_glrlm_GrayLevelVariance,

square_glrlm_HighGrayLevelRunEmphasis,

square_glrlm_LongRunEmphasis,

square_glrlm_LongRunHighGrayLevelEmphasis,

square_glrlm_LongRunLowGrayLevelEmphasis,

square_glrlm_LowGrayLevelRunEmphasis,

square_glrlm_RunEntropy,

square_glrlm_RunLengthNonUniformity,

square_glrlm_RunLengthNonUniformityNormalized,

square_glrlm_RunPercentage,

square_glrlm_RunVariance,

square_glrlm_ShortRunEmphasis,

square_glrlm_ShortRunHighGrayLevelEmphasis,

square_glrlm_ShortRunLowGrayLevelEmphasis,

square_glszm_GrayLevelNonUniformity,

square_glszm_GrayLevelNonUniformityNormalized,

square_glszm_GrayLevelVariance,

square_glszm_HighGrayLevelZoneEmphasis,

square_glszm_LargeAreaEmphasis,

square_glszm_LargeAreaHighGrayLevelEmphasis,

square_glszm_LargeAreaLowGrayLevelEmphasis,

square_glszm_LowGrayLevelZoneEmphasis,

square_glszm_SizeZoneNonUniformity,

square_glszm_SizeZoneNonUniformityNormalized,

square_glszm_SmallAreaEmphasis,

square_glszm_SmallAreaHighGrayLevelEmphasis,

square_glszm_SmallAreaLowGrayLevelEmphasis,

square_glszm_ZoneEntropy,

square_glszm_ZonePercentage,

square_glszm_ZoneVariance,

square_ngtdm_Busyness,

square_ngtdm_Coarseness,

square_ngtdm_Complexity,

square_ngtdm_Contrast,

square_ngtdm_Strength,

squareroot_firstorder_10Percentile,

squareroot_firstorder_90Percentile,

squareroot_firstorder_Energy,

squareroot_firstorder_Entropy,

squareroot_firstorder_InterquartileRange,

squareroot_firstorder_Kurtosis,

squareroot_firstorder_Maximum,

squareroot_firstorder_Mean,

squareroot_firstorder_MeanAbsoluteDeviation,

squareroot_firstorder_Median,

squareroot_firstorder_Minimum,

squareroot_firstorder_Range,

squareroot_firstorder_RobustMeanAbsoluteDeviation,

squareroot_firstorder_RootMeanSquared,

squareroot_firstorder_Skewness,

squareroot_firstorder_TotalEnergy,

squareroot_firstorder_Uniformity,

squareroot_firstorder_Variance,

squareroot_glcm_Autocorrelation,

squareroot_glcm_ClusterProminence,

squareroot_glcm_ClusterShade,

squareroot_glcm_ClusterTendency,

squareroot_glcm_Contrast,

squareroot_glcm_Correlation,

squareroot_glcm_DifferenceAverage,

squareroot_glcm_DifferenceEntropy,

squareroot_glcm_DifferenceVariance,

squareroot_glcm_Id,

squareroot_glcm_Idm,

squareroot_glcm_Idmn,

squareroot_glcm_Idn,

squareroot_glcm_Imc1,

squareroot_glcm_Imc2,

squareroot_glcm_InverseVariance,

squareroot_glcm_JointAverage,

squareroot_glcm_JointEnergy,

squareroot_glcm_JointEntropy,

squareroot_glcm_MaximumProbability,

squareroot_glcm_SumEntropy,

squareroot_glcm_SumSquares,

squareroot_gldm_DependenceEntropy,

squareroot_gldm_DependenceNonUniformity,

squareroot_gldm_DependenceNonUniformityNormalized,

squareroot_gldm_DependenceVariance,

squareroot_gldm_GrayLevelNonUniformity,

squareroot_gldm_GrayLevelVariance,

squareroot_gldm_HighGrayLevelEmphasis,

squareroot_gldm_LargeDependenceEmphasis,

squareroot_gldm_LargeDependenceHighGrayLevelEmphasis,

squareroot_gldm_LargeDependenceLowGrayLevelEmphasis,

squareroot_gldm_LowGrayLevelEmphasis,

squareroot_gldm_SmallDependenceEmphasis,

squareroot_gldm_SmallDependenceHighGrayLevelEmphasis,

squareroot_gldm_SmallDependenceLowGrayLevelEmphasis,

squareroot_glrlm_GrayLevelNonUniformity,

squareroot_glrlm_GrayLevelNonUniformityNormalized,

squareroot_glrlm_GrayLevelVariance,

squareroot_glrlm_HighGrayLevelRunEmphasis,

squareroot_glrlm_LongRunEmphasis,

squareroot_glrlm_LongRunHighGrayLevelEmphasis,

squareroot_glrlm_LongRunLowGrayLevelEmphasis,

squareroot_glrlm_LowGrayLevelRunEmphasis,

squareroot_glrlm_RunEntropy,

squareroot_glrlm_RunLengthNonUniformity,

squareroot_glrlm_RunLengthNonUniformityNormalized,

squareroot_glrlm_RunPercentage,

squareroot_glrlm_RunVariance,

squareroot_glrlm_ShortRunEmphasis,

squareroot_glrlm_ShortRunHighGrayLevelEmphasis,

squareroot_glrlm_ShortRunLowGrayLevelEmphasis,

squareroot_glszm_GrayLevelNonUniformity,

squareroot_glszm_GrayLevelNonUniformityNormalized,

squareroot_glszm_GrayLevelVariance,

squareroot_glszm_HighGrayLevelZoneEmphasis,

squareroot_glszm_LargeAreaEmphasis,

squareroot_glszm_LargeAreaHighGrayLevelEmphasis,

squareroot_glszm_LargeAreaLowGrayLevelEmphasis,

squareroot_glszm_LowGrayLevelZoneEmphasis,

squareroot_glszm_SizeZoneNonUniformity,

squareroot_glszm_SizeZoneNonUniformityNormalized,

squareroot_glszm_SmallAreaEmphasis,

squareroot_glszm_SmallAreaHighGrayLevelEmphasis,

squareroot_glszm_SmallAreaLowGrayLevelEmphasis,

squareroot_glszm_ZoneEntropy,

squareroot_glszm_ZonePercentage,

squareroot_glszm_ZoneVariance,

squareroot_ngtdm_Busyness,

squareroot_ngtdm_Coarseness,

squareroot_ngtdm_Complexity,

squareroot_ngtdm_Contrast,

squareroot_ngtdm_Strength,

wavelet_HHH_firstorder_10Percentile,

wavelet_HHH_firstorder_90Percentile,

wavelet_HHH_firstorder_Energy,

wavelet_HHH_firstorder_Entropy,

wavelet_HHH_firstorder_InterquartileRange,

wavelet_HHH_firstorder_Kurtosis,

wavelet_HHH_firstorder_Maximum,

wavelet_HHH_firstorder_Mean,

wavelet_HHH_firstorder_MeanAbsoluteDeviation,

wavelet_HHH_firstorder_Median,

wavelet_HHH_firstorder_Minimum,

wavelet_HHH_firstorder_Range,

wavelet_HHH_firstorder_RobustMeanAbsoluteDeviation,

wavelet_HHH_firstorder_RootMeanSquared,

wavelet_HHH_firstorder_Skewness,

wavelet_HHH_firstorder_TotalEnergy,

wavelet_HHH_firstorder_Uniformity,

wavelet_HHH_firstorder_Variance,

wavelet_HHH_glcm_Autocorrelation,

wavelet_HHH_glcm_ClusterProminence,

wavelet_HHH_glcm_ClusterShade,

wavelet_HHH_glcm_ClusterTendency,

wavelet_HHH_glcm_Contrast,

wavelet_HHH_glcm_Correlation,

wavelet_HHH_glcm_DifferenceAverage,

wavelet_HHH_glcm_DifferenceEntropy,

wavelet_HHH_glcm_DifferenceVariance,

wavelet_HHH_glcm_Id,

wavelet_HHH_glcm_Idm,

wavelet_HHH_glcm_Idmn,

wavelet_HHH_glcm_Idn,

wavelet_HHH_glcm_Imc1,

wavelet_HHH_glcm_Imc2,

wavelet_HHH_glcm_InverseVariance,

wavelet_HHH_glcm_JointAverage,

wavelet_HHH_glcm_JointEnergy,

wavelet_HHH_glcm_JointEntropy,

wavelet_HHH_glcm_MaximumProbability,

wavelet_HHH_glcm_SumEntropy,

wavelet_HHH_glcm_SumSquares,

wavelet_HHH_gldm_DependenceEntropy,

wavelet_HHH_gldm_DependenceNonUniformity,

wavelet_HHH_gldm_DependenceNonUniformityNormalized,

wavelet_HHH_gldm_DependenceVariance,

wavelet_HHH_gldm_GrayLevelNonUniformity,

wavelet_HHH_gldm_GrayLevelVariance,

wavelet_HHH_gldm_HighGrayLevelEmphasis,

wavelet_HHH_gldm_LargeDependenceEmphasis,

wavelet_HHH_gldm_LargeDependenceHighGrayLevelEmphasis,

wavelet_HHH_gldm_LargeDependenceLowGrayLevelEmphasis,

wavelet_HHH_gldm_LowGrayLevelEmphasis,

wavelet_HHH_gldm_SmallDependenceEmphasis,

wavelet_HHH_gldm_SmallDependenceHighGrayLevelEmphasis,

wavelet_HHH_gldm_SmallDependenceLowGrayLevelEmphasis,

wavelet_HHH_glrlm_GrayLevelNonUniformity,

wavelet_HHH_glrlm_GrayLevelNonUniformityNormalized,

wavelet_HHH_glrlm_GrayLevelVariance,

wavelet_HHH_glrlm_HighGrayLevelRunEmphasis,

wavelet_HHH_glrlm_LongRunEmphasis,

wavelet_HHH_glrlm_LongRunHighGrayLevelEmphasis,

wavelet_HHH_glrlm_LongRunLowGrayLevelEmphasis,

wavelet_HHH_glrlm_LowGrayLevelRunEmphasis,

wavelet_HHH_glrlm_RunEntropy,

wavelet_HHH_glrlm_RunLengthNonUniformity,

wavelet_HHH_glrlm_RunLengthNonUniformityNormalized,

wavelet_HHH_glrlm_RunPercentage,

wavelet_HHH_glrlm_RunVariance,

wavelet_HHH_glrlm_ShortRunEmphasis,

wavelet_HHH_glrlm_ShortRunHighGrayLevelEmphasis,

wavelet_HHH_glrlm_ShortRunLowGrayLevelEmphasis,

wavelet_HHH_glszm_GrayLevelNonUniformity,

wavelet_HHH_glszm_GrayLevelNonUniformityNormalized,

wavelet_HHH_glszm_GrayLevelVariance,

wavelet_HHH_glszm_HighGrayLevelZoneEmphasis,

wavelet_HHH_glszm_LargeAreaEmphasis,

wavelet_HHH_glszm_LargeAreaHighGrayLevelEmphasis,

wavelet_HHH_glszm_LargeAreaLowGrayLevelEmphasis,

wavelet_HHH_glszm_LowGrayLevelZoneEmphasis,

wavelet_HHH_glszm_SizeZoneNonUniformity,

wavelet_HHH_glszm_SizeZoneNonUniformityNormalized,

wavelet_HHH_glszm_SmallAreaEmphasis,

wavelet_HHH_glszm_SmallAreaHighGrayLevelEmphasis,

wavelet_HHH_glszm_SmallAreaLowGrayLevelEmphasis,

wavelet_HHH_glszm_ZoneEntropy,

wavelet_HHH_glszm_ZonePercentage,

wavelet_HHH_glszm_ZoneVariance,

wavelet_HHH_ngtdm_Busyness,

wavelet_HHH_ngtdm_Coarseness,

wavelet_HHH_ngtdm_Complexity,

wavelet_HHH_ngtdm_Contrast,

wavelet_HHH_ngtdm_Strength,

wavelet_HHL_firstorder_10Percentile,

wavelet_HHL_firstorder_90Percentile,

wavelet_HHL_firstorder_Energy,

wavelet_HHL_firstorder_Entropy,

wavelet_HHL_firstorder_InterquartileRange,

wavelet_HHL_firstorder_Kurtosis,

wavelet_HHL_firstorder_Maximum,

wavelet_HHL_firstorder_Mean,

wavelet_HHL_firstorder_MeanAbsoluteDeviation,

wavelet_HHL_firstorder_Median,

wavelet_HHL_firstorder_Minimum,

wavelet_HHL_firstorder_Range,

wavelet_HHL_firstorder_RobustMeanAbsoluteDeviation,

wavelet_HHL_firstorder_RootMeanSquared,

wavelet_HHL_firstorder_Skewness,

wavelet_HHL_firstorder_TotalEnergy,

wavelet_HHL_firstorder_Uniformity,

wavelet_HHL_firstorder_Variance,

wavelet_HHL_glcm_Autocorrelation,

wavelet_HHL_glcm_ClusterProminence,

wavelet_HHL_glcm_ClusterShade,

wavelet_HHL_glcm_ClusterTendency,

wavelet_HHL_glcm_Contrast,

wavelet_HHL_glcm_Correlation,

wavelet_HHL_glcm_DifferenceAverage,

wavelet_HHL_glcm_DifferenceEntropy,

wavelet_HHL_glcm_DifferenceVariance,

wavelet_HHL_glcm_Id,

wavelet_HHL_glcm_Idm,

wavelet_HHL_glcm_Idmn,

wavelet_HHL_glcm_Idn,

wavelet_HHL_glcm_Imc1,

wavelet_HHL_glcm_Imc2,

wavelet_HHL_glcm_InverseVariance,

wavelet_HHL_glcm_JointAverage,

wavelet_HHL_glcm_JointEnergy,

wavelet_HHL_glcm_JointEntropy,

wavelet_HHL_glcm_MaximumProbability,

wavelet_HHL_glcm_SumEntropy,

wavelet_HHL_glcm_SumSquares,

wavelet_HHL_gldm_DependenceEntropy,

wavelet_HHL_gldm_DependenceNonUniformity,

wavelet_HHL_gldm_DependenceNonUniformityNormalized,

wavelet_HHL_gldm_DependenceVariance,

wavelet_HHL_gldm_GrayLevelNonUniformity,

wavelet_HHL_gldm_GrayLevelVariance,

wavelet_HHL_gldm_HighGrayLevelEmphasis,

wavelet_HHL_gldm_LargeDependenceEmphasis,

wavelet_HHL_gldm_LargeDependenceHighGrayLevelEmphasis,

wavelet_HHL_gldm_LargeDependenceLowGrayLevelEmphasis,

wavelet_HHL_gldm_LowGrayLevelEmphasis,

wavelet_HHL_gldm_SmallDependenceEmphasis,

wavelet_HHL_gldm_SmallDependenceHighGrayLevelEmphasis,

wavelet_HHL_gldm_SmallDependenceLowGrayLevelEmphasis,

wavelet_HHL_glrlm_GrayLevelNonUniformity,

wavelet_HHL_glrlm_GrayLevelNonUniformityNormalized,

wavelet_HHL_glrlm_GrayLevelVariance,

wavelet_HHL_glrlm_HighGrayLevelRunEmphasis,

wavelet_HHL_glrlm_LongRunEmphasis,

wavelet_HHL_glrlm_LongRunHighGrayLevelEmphasis,

wavelet_HHL_glrlm_LongRunLowGrayLevelEmphasis,

wavelet_HHL_glrlm_LowGrayLevelRunEmphasis,

wavelet_HHL_glrlm_RunEntropy,

wavelet_HHL_glrlm_RunLengthNonUniformity,

wavelet_HHL_glrlm_RunLengthNonUniformityNormalized,

wavelet_HHL_glrlm_RunPercentage,

wavelet_HHL_glrlm_RunVariance,

wavelet_HHL_glrlm_ShortRunEmphasis,

wavelet_HHL_glrlm_ShortRunHighGrayLevelEmphasis,

wavelet_HHL_glrlm_ShortRunLowGrayLevelEmphasis,

wavelet_HHL_glszm_GrayLevelNonUniformity,

wavelet_HHL_glszm_GrayLevelNonUniformityNormalized,

wavelet_HHL_glszm_GrayLevelVariance,

wavelet_HHL_glszm_HighGrayLevelZoneEmphasis,

wavelet_HHL_glszm_LargeAreaEmphasis,

wavelet_HHL_glszm_LargeAreaHighGrayLevelEmphasis,

wavelet_HHL_glszm_LargeAreaLowGrayLevelEmphasis,

wavelet_HHL_glszm_LowGrayLevelZoneEmphasis,

wavelet_HHL_glszm_SizeZoneNonUniformity,

wavelet_HHL_glszm_SizeZoneNonUniformityNormalized,

wavelet_HHL_glszm_SmallAreaEmphasis,

wavelet_HHL_glszm_SmallAreaHighGrayLevelEmphasis,

wavelet_HHL_glszm_SmallAreaLowGrayLevelEmphasis,

wavelet_HHL_glszm_ZoneEntropy,

wavelet_HHL_glszm_ZonePercentage,

wavelet_HHL_glszm_ZoneVariance,

wavelet_HHL_ngtdm_Busyness,

wavelet_HHL_ngtdm_Coarseness,

wavelet_HHL_ngtdm_Complexity,

wavelet_HHL_ngtdm_Contrast,

wavelet_HHL_ngtdm_Strength,

wavelet_HLH_firstorder_10Percentile,

wavelet_HLH_firstorder_90Percentile,

wavelet_HLH_firstorder_Energy,

wavelet_HLH_firstorder_Entropy,

wavelet_HLH_firstorder_InterquartileRange,

wavelet_HLH_firstorder_Kurtosis,

wavelet_HLH_firstorder_Maximum,

wavelet_HLH_firstorder_Mean,

wavelet_HLH_firstorder_MeanAbsoluteDeviation,

wavelet_HLH_firstorder_Median,

wavelet_HLH_firstorder_Minimum,

wavelet_HLH_firstorder_Range,

wavelet_HLH_firstorder_RobustMeanAbsoluteDeviation,

wavelet_HLH_firstorder_RootMeanSquared,

wavelet_HLH_firstorder_Skewness,

wavelet_HLH_firstorder_TotalEnergy,

wavelet_HLH_firstorder_Uniformity,

wavelet_HLH_firstorder_Variance,

wavelet_HLH_glcm_Autocorrelation,

wavelet_HLH_glcm_ClusterProminence,

wavelet_HLH_glcm_ClusterShade,

wavelet_HLH_glcm_ClusterTendency,

wavelet_HLH_glcm_Contrast,

wavelet_HLH_glcm_Correlation,

wavelet_HLH_glcm_DifferenceAverage,

wavelet_HLH_glcm_DifferenceEntropy,

wavelet_HLH_glcm_DifferenceVariance,

wavelet_HLH_glcm_Id,

wavelet_HLH_glcm_Idm,

wavelet_HLH_glcm_Idmn,

wavelet_HLH_glcm_Idn,

wavelet_HLH_glcm_Imc1,

wavelet_HLH_glcm_Imc2,

wavelet_HLH_glcm_InverseVariance,

wavelet_HLH_glcm_JointAverage,

wavelet_HLH_glcm_JointEnergy,

wavelet_HLH_glcm_JointEntropy,

wavelet_HLH_glcm_MaximumProbability,

wavelet_HLH_glcm_SumEntropy,

wavelet_HLH_glcm_SumSquares,

wavelet_HLH_gldm_DependenceEntropy,

wavelet_HLH_gldm_DependenceNonUniformity,

wavelet_HLH_gldm_DependenceNonUniformityNormalized,

wavelet_HLH_gldm_DependenceVariance,

wavelet_HLH_gldm_GrayLevelNonUniformity,

wavelet_HLH_gldm_GrayLevelVariance,

wavelet_HLH_gldm_HighGrayLevelEmphasis,

wavelet_HLH_gldm_LargeDependenceEmphasis,

wavelet_HLH_gldm_LargeDependenceHighGrayLevelEmphasis,

wavelet_HLH_gldm_LargeDependenceLowGrayLevelEmphasis,

wavelet_HLH_gldm_LowGrayLevelEmphasis,

wavelet_HLH_gldm_SmallDependenceEmphasis,

wavelet_HLH_gldm_SmallDependenceHighGrayLevelEmphasis,

wavelet_HLH_gldm_SmallDependenceLowGrayLevelEmphasis,

wavelet_HLH_glrlm_GrayLevelNonUniformity,

wavelet_HLH_glrlm_GrayLevelNonUniformityNormalized,

wavelet_HLH_glrlm_GrayLevelVariance,

wavelet_HLH_glrlm_HighGrayLevelRunEmphasis,

wavelet_HLH_glrlm_LongRunEmphasis,

wavelet_HLH_glrlm_LongRunHighGrayLevelEmphasis,

wavelet_HLH_glrlm_LongRunLowGrayLevelEmphasis,

wavelet_HLH_glrlm_LowGrayLevelRunEmphasis,

wavelet_HLH_glrlm_RunEntropy,

wavelet_HLH_glrlm_RunLengthNonUniformity,

wavelet_HLH_glrlm_RunLengthNonUniformityNormalized,

wavelet_HLH_glrlm_RunPercentage,

wavelet_HLH_glrlm_RunVariance,

wavelet_HLH_glrlm_ShortRunEmphasis,

wavelet_HLH_glrlm_ShortRunHighGrayLevelEmphasis,

wavelet_HLH_glrlm_ShortRunLowGrayLevelEmphasis,

wavelet_HLH_glszm_GrayLevelNonUniformity,

wavelet_HLH_glszm_GrayLevelNonUniformityNormalized,

wavelet_HLH_glszm_GrayLevelVariance,

wavelet_HLH_glszm_HighGrayLevelZoneEmphasis,

wavelet_HLH_glszm_LargeAreaEmphasis,

wavelet_HLH_glszm_LargeAreaHighGrayLevelEmphasis,

wavelet_HLH_glszm_LargeAreaLowGrayLevelEmphasis,

wavelet_HLH_glszm_LowGrayLevelZoneEmphasis,

wavelet_HLH_glszm_SizeZoneNonUniformity,

wavelet_HLH_glszm_SizeZoneNonUniformityNormalized,

wavelet_HLH_glszm_SmallAreaEmphasis,

wavelet_HLH_glszm_SmallAreaHighGrayLevelEmphasis,

wavelet_HLH_glszm_SmallAreaLowGrayLevelEmphasis,

wavelet_HLH_glszm_ZoneEntropy,

wavelet_HLH_glszm_ZonePercentage,

wavelet_HLH_glszm_ZoneVariance,

wavelet_HLH_ngtdm_Busyness,

wavelet_HLH_ngtdm_Coarseness,

wavelet_HLH_ngtdm_Complexity,

wavelet_HLH_ngtdm_Contrast,

wavelet_HLH_ngtdm_Strength,

wavelet_HLL_firstorder_10Percentile,

wavelet_HLL_firstorder_90Percentile,

wavelet_HLL_firstorder_Energy,

wavelet_HLL_firstorder_Entropy,

wavelet_HLL_firstorder_InterquartileRange,

wavelet_HLL_firstorder_Kurtosis,

wavelet_HLL_firstorder_Maximum,

wavelet_HLL_firstorder_Mean,

wavelet_HLL_firstorder_MeanAbsoluteDeviation,

wavelet_HLL_firstorder_Median,

wavelet_HLL_firstorder_Minimum,

wavelet_HLL_firstorder_Range,

wavelet_HLL_firstorder_RobustMeanAbsoluteDeviation,

wavelet_HLL_firstorder_RootMeanSquared,

wavelet_HLL_firstorder_Skewness,

wavelet_HLL_firstorder_TotalEnergy,

wavelet_HLL_firstorder_Uniformity,

wavelet_HLL_firstorder_Variance,

wavelet_HLL_glcm_Autocorrelation,

wavelet_HLL_glcm_ClusterProminence,

wavelet_HLL_glcm_ClusterShade,

wavelet_HLL_glcm_ClusterTendency,

wavelet_HLL_glcm_Contrast,

wavelet_HLL_glcm_Correlation,

wavelet_HLL_glcm_DifferenceAverage,

wavelet_HLL_glcm_DifferenceEntropy,

wavelet_HLL_glcm_DifferenceVariance,

wavelet_HLL_glcm_Id,

wavelet_HLL_glcm_Idm,

wavelet_HLL_glcm_Idmn,

wavelet_HLL_glcm_Idn,

wavelet_HLL_glcm_Imc1,

wavelet_HLL_glcm_Imc2,

wavelet_HLL_glcm_InverseVariance,

wavelet_HLL_glcm_JointAverage,

wavelet_HLL_glcm_JointEnergy,

wavelet_HLL_glcm_JointEntropy,

wavelet_HLL_glcm_MaximumProbability,

wavelet_HLL_glcm_SumEntropy,

wavelet_HLL_glcm_SumSquares,

wavelet_HLL_gldm_DependenceEntropy,

wavelet_HLL_gldm_DependenceNonUniformity,

wavelet_HLL_gldm_DependenceNonUniformityNormalized,

wavelet_HLL_gldm_DependenceVariance,

wavelet_HLL_gldm_GrayLevelNonUniformity,

wavelet_HLL_gldm_GrayLevelVariance,

wavelet_HLL_gldm_HighGrayLevelEmphasis,

wavelet_HLL_gldm_LargeDependenceEmphasis,

wavelet_HLL_gldm_LargeDependenceHighGrayLevelEmphasis,

wavelet_HLL_gldm_LargeDependenceLowGrayLevelEmphasis,

wavelet_HLL_gldm_LowGrayLevelEmphasis,

wavelet_HLL_gldm_SmallDependenceEmphasis,

wavelet_HLL_gldm_SmallDependenceHighGrayLevelEmphasis,

wavelet_HLL_gldm_SmallDependenceLowGrayLevelEmphasis,

wavelet_HLL_glrlm_GrayLevelNonUniformity,

wavelet_HLL_glrlm_GrayLevelNonUniformityNormalized,

wavelet_HLL_glrlm_GrayLevelVariance,

wavelet_HLL_glrlm_HighGrayLevelRunEmphasis,

wavelet_HLL_glrlm_LongRunEmphasis,

wavelet_HLL_glrlm_LongRunHighGrayLevelEmphasis,

wavelet_HLL_glrlm_LongRunLowGrayLevelEmphasis,

wavelet_HLL_glrlm_LowGrayLevelRunEmphasis,

wavelet_HLL_glrlm_RunEntropy,

wavelet_HLL_glrlm_RunLengthNonUniformity,

wavelet_HLL_glrlm_RunLengthNonUniformityNormalized,

wavelet_HLL_glrlm_RunPercentage,

wavelet_HLL_glrlm_RunVariance,

wavelet_HLL_glrlm_ShortRunEmphasis,

wavelet_HLL_glrlm_ShortRunHighGrayLevelEmphasis,

wavelet_HLL_glrlm_ShortRunLowGrayLevelEmphasis,

wavelet_HLL_glszm_GrayLevelNonUniformity,

wavelet_HLL_glszm_GrayLevelNonUniformityNormalized,

wavelet_HLL_glszm_GrayLevelVariance,

wavelet_HLL_glszm_HighGrayLevelZoneEmphasis,

wavelet_HLL_glszm_LargeAreaEmphasis,

wavelet_HLL_glszm_LargeAreaHighGrayLevelEmphasis,

wavelet_HLL_glszm_LargeAreaLowGrayLevelEmphasis,

wavelet_HLL_glszm_LowGrayLevelZoneEmphasis,

wavelet_HLL_glszm_SizeZoneNonUniformity,

wavelet_HLL_glszm_SizeZoneNonUniformityNormalized,

wavelet_HLL_glszm_SmallAreaEmphasis,

wavelet_HLL_glszm_SmallAreaHighGrayLevelEmphasis,

wavelet_HLL_glszm_SmallAreaLowGrayLevelEmphasis,

wavelet_HLL_glszm_ZoneEntropy,

wavelet_HLL_glszm_ZonePercentage,

wavelet_HLL_glszm_ZoneVariance,

wavelet_HLL_ngtdm_Busyness,

wavelet_HLL_ngtdm_Coarseness,

wavelet_HLL_ngtdm_Complexity,

wavelet_HLL_ngtdm_Contrast,

wavelet_HLL_ngtdm_Strength,

wavelet_LHH_firstorder_10Percentile,

wavelet_LHH_firstorder_90Percentile,

wavelet_LHH_firstorder_Energy,

wavelet_LHH_firstorder_Entropy,

wavelet_LHH_firstorder_InterquartileRange,

wavelet_LHH_firstorder_Kurtosis,

wavelet_LHH_firstorder_Maximum,

wavelet_LHH_firstorder_Mean,

wavelet_LHH_firstorder_MeanAbsoluteDeviation,

wavelet_LHH_firstorder_Median,

wavelet_LHH_firstorder_Minimum,

wavelet_LHH_firstorder_Range,

wavelet_LHH_firstorder_RobustMeanAbsoluteDeviation,

wavelet_LHH_firstorder_RootMeanSquared,

wavelet_LHH_firstorder_Skewness,

wavelet_LHH_firstorder_TotalEnergy,

wavelet_LHH_firstorder_Uniformity,

wavelet_LHH_firstorder_Variance,

wavelet_LHH_glcm_Autocorrelation,

wavelet_LHH_glcm_ClusterProminence,

wavelet_LHH_glcm_ClusterShade,

wavelet_LHH_glcm_ClusterTendency,

wavelet_LHH_glcm_Contrast,

wavelet_LHH_glcm_Correlation,

wavelet_LHH_glcm_DifferenceAverage,

wavelet_LHH_glcm_DifferenceEntropy,

wavelet_LHH_glcm_DifferenceVariance,

wavelet_LHH_glcm_Id,

wavelet_LHH_glcm_Idm,

wavelet_LHH_glcm_Idmn,

wavelet_LHH_glcm_Idn,

wavelet_LHH_glcm_Imc1,

wavelet_LHH_glcm_Imc2,

wavelet_LHH_glcm_InverseVariance,

wavelet_LHH_glcm_JointAverage,

wavelet_LHH_glcm_JointEnergy,

wavelet_LHH_glcm_JointEntropy,

wavelet_LHH_glcm_MaximumProbability,

wavelet_LHH_glcm_SumEntropy,

wavelet_LHH_glcm_SumSquares,

wavelet_LHH_gldm_DependenceEntropy,

wavelet_LHH_gldm_DependenceNonUniformity,

wavelet_LHH_gldm_DependenceNonUniformityNormalized,

wavelet_LHH_gldm_DependenceVariance,

wavelet_LHH_gldm_GrayLevelNonUniformity,

wavelet_LHH_gldm_GrayLevelVariance,

wavelet_LHH_gldm_HighGrayLevelEmphasis,

wavelet_LHH_gldm_LargeDependenceEmphasis,

wavelet_LHH_gldm_LargeDependenceHighGrayLevelEmphasis,

wavelet_LHH_gldm_LargeDependenceLowGrayLevelEmphasis,

wavelet_LHH_gldm_LowGrayLevelEmphasis,

wavelet_LHH_gldm_SmallDependenceEmphasis,

wavelet_LHH_gldm_SmallDependenceHighGrayLevelEmphasis,

wavelet_LHH_gldm_SmallDependenceLowGrayLevelEmphasis,

wavelet_LHH_glrlm_GrayLevelNonUniformity,

wavelet_LHH_glrlm_GrayLevelNonUniformityNormalized,

wavelet_LHH_glrlm_GrayLevelVariance,

wavelet_LHH_glrlm_HighGrayLevelRunEmphasis,

wavelet_LHH_glrlm_LongRunEmphasis,

wavelet_LHH_glrlm_LongRunHighGrayLevelEmphasis,

wavelet_LHH_glrlm_LongRunLowGrayLevelEmphasis,

wavelet_LHH_glrlm_LowGrayLevelRunEmphasis,

wavelet_LHH_glrlm_RunEntropy,

wavelet_LHH_glrlm_RunLengthNonUniformity,

wavelet_LHH_glrlm_RunLengthNonUniformityNormalized,

wavelet_LHH_glrlm_RunPercentage,

wavelet_LHH_glrlm_RunVariance,

wavelet_LHH_glrlm_ShortRunEmphasis,

wavelet_LHH_glrlm_ShortRunHighGrayLevelEmphasis,

wavelet_LHH_glrlm_ShortRunLowGrayLevelEmphasis,

wavelet_LHH_glszm_GrayLevelNonUniformity,

wavelet_LHH_glszm_GrayLevelNonUniformityNormalized,

wavelet_LHH_glszm_GrayLevelVariance,

wavelet_LHH_glszm_HighGrayLevelZoneEmphasis,

wavelet_LHH_glszm_LargeAreaEmphasis,

wavelet_LHH_glszm_LargeAreaHighGrayLevelEmphasis,

wavelet_LHH_glszm_LargeAreaLowGrayLevelEmphasis,

wavelet_LHH_glszm_LowGrayLevelZoneEmphasis,

wavelet_LHH_glszm_SizeZoneNonUniformity,

wavelet_LHH_glszm_SizeZoneNonUniformityNormalized,

wavelet_LHH_glszm_SmallAreaEmphasis,

wavelet_LHH_glszm_SmallAreaHighGrayLevelEmphasis,

wavelet_LHH_glszm_SmallAreaLowGrayLevelEmphasis,

wavelet_LHH_glszm_ZoneEntropy,

wavelet_LHH_glszm_ZonePercentage,

wavelet_LHH_glszm_ZoneVariance,

wavelet_LHH_ngtdm_Busyness,

wavelet_LHH_ngtdm_Coarseness,

wavelet_LHH_ngtdm_Complexity,

wavelet_LHH_ngtdm_Contrast,

wavelet_LHH_ngtdm_Strength,

wavelet_LHL_firstorder_10Percentile,

wavelet_LHL_firstorder_90Percentile,

wavelet_LHL_firstorder_Energy,

wavelet_LHL_firstorder_Entropy,

wavelet_LHL_firstorder_InterquartileRange,

wavelet_LHL_firstorder_Kurtosis,

wavelet_LHL_firstorder_Maximum,

wavelet_LHL_firstorder_Mean,

wavelet_LHL_firstorder_MeanAbsoluteDeviation,

wavelet_LHL_firstorder_Median,

wavelet_LHL_firstorder_Minimum,

wavelet_LHL_firstorder_Range,

wavelet_LHL_firstorder_RobustMeanAbsoluteDeviation,

wavelet_LHL_firstorder_RootMeanSquared,

wavelet_LHL_firstorder_Skewness,

wavelet_LHL_firstorder_TotalEnergy,

wavelet_LHL_firstorder_Uniformity,

wavelet_LHL_firstorder_Variance,

wavelet_LHL_glcm_Autocorrelation,

wavelet_LHL_glcm_ClusterProminence,

wavelet_LHL_glcm_ClusterShade,

wavelet_LHL_glcm_ClusterTendency,

wavelet_LHL_glcm_Contrast,

wavelet_LHL_glcm_Correlation,

wavelet_LHL_glcm_DifferenceAverage,

wavelet_LHL_glcm_DifferenceEntropy,

wavelet_LHL_glcm_DifferenceVariance,

wavelet_LHL_glcm_Id,

wavelet_LHL_glcm_Idm,

wavelet_LHL_glcm_Idmn,

wavelet_LHL_glcm_Idn,

wavelet_LHL_glcm_Imc1,

wavelet_LHL_glcm_Imc2,

wavelet_LHL_glcm_InverseVariance,

wavelet_LHL_glcm_JointAverage,

wavelet_LHL_glcm_JointEnergy,

wavelet_LHL_glcm_JointEntropy,

wavelet_LHL_glcm_MaximumProbability,

wavelet_LHL_glcm_SumEntropy,

wavelet_LHL_glcm_SumSquares,

wavelet_LHL_gldm_DependenceEntropy,

wavelet_LHL_gldm_DependenceNonUniformity,

wavelet_LHL_gldm_DependenceNonUniformityNormalized,

wavelet_LHL_gldm_DependenceVariance,

wavelet_LHL_gldm_GrayLevelNonUniformity,

wavelet_LHL_gldm_GrayLevelVariance,

wavelet_LHL_gldm_HighGrayLevelEmphasis,

wavelet_LHL_gldm_LargeDependenceEmphasis,

wavelet_LHL_gldm_LargeDependenceHighGrayLevelEmphasis,

wavelet_LHL_gldm_LargeDependenceLowGrayLevelEmphasis,

wavelet_LHL_gldm_LowGrayLevelEmphasis,

wavelet_LHL_gldm_SmallDependenceEmphasis,

wavelet_LHL_gldm_SmallDependenceHighGrayLevelEmphasis,

wavelet_LHL_gldm_SmallDependenceLowGrayLevelEmphasis,

wavelet_LHL_glrlm_GrayLevelNonUniformity,

wavelet_LHL_glrlm_GrayLevelNonUniformityNormalized,

wavelet_LHL_glrlm_GrayLevelVariance,

wavelet_LHL_glrlm_HighGrayLevelRunEmphasis,

wavelet_LHL_glrlm_LongRunEmphasis,

wavelet_LHL_glrlm_LongRunHighGrayLevelEmphasis,

wavelet_LHL_glrlm_LongRunLowGrayLevelEmphasis,

wavelet_LHL_glrlm_LowGrayLevelRunEmphasis,

wavelet_LHL_glrlm_RunEntropy,

wavelet_LHL_glrlm_RunLengthNonUniformity,

wavelet_LHL_glrlm_RunLengthNonUniformityNormalized,

wavelet_LHL_glrlm_RunPercentage,

wavelet_LHL_glrlm_RunVariance,

wavelet_LHL_glrlm_ShortRunEmphasis,

wavelet_LHL_glrlm_ShortRunHighGrayLevelEmphasis,

wavelet_LHL_glrlm_ShortRunLowGrayLevelEmphasis,

wavelet_LHL_glszm_GrayLevelNonUniformity,

wavelet_LHL_glszm_GrayLevelNonUniformityNormalized,

wavelet_LHL_glszm_GrayLevelVariance,

wavelet_LHL_glszm_HighGrayLevelZoneEmphasis,

wavelet_LHL_glszm_LargeAreaEmphasis,

wavelet_LHL_glszm_LargeAreaHighGrayLevelEmphasis,

wavelet_LHL_glszm_LargeAreaLowGrayLevelEmphasis,

wavelet_LHL_glszm_LowGrayLevelZoneEmphasis,

wavelet_LHL_glszm_SizeZoneNonUniformity,

wavelet_LHL_glszm_SizeZoneNonUniformityNormalized,

wavelet_LHL_glszm_SmallAreaEmphasis,

wavelet_LHL_glszm_SmallAreaHighGrayLevelEmphasis,

wavelet_LHL_glszm_SmallAreaLowGrayLevelEmphasis,

wavelet_LHL_glszm_ZoneEntropy,

wavelet_LHL_glszm_ZonePercentage,

wavelet_LHL_glszm_ZoneVariance,

wavelet_LHL_ngtdm_Busyness,

wavelet_LHL_ngtdm_Coarseness,

wavelet_LHL_ngtdm_Complexity,

wavelet_LHL_ngtdm_Contrast,

wavelet_LHL_ngtdm_Strength,

wavelet_LLH_firstorder_10Percentile,

wavelet_LLH_firstorder_90Percentile,

wavelet_LLH_firstorder_Energy,

wavelet_LLH_firstorder_Entropy,

wavelet_LLH_firstorder_InterquartileRange,

wavelet_LLH_firstorder_Kurtosis,

wavelet_LLH_firstorder_Maximum,

wavelet_LLH_firstorder_Mean,

wavelet_LLH_firstorder_MeanAbsoluteDeviation,

wavelet_LLH_firstorder_Median,

wavelet_LLH_firstorder_Minimum,

wavelet_LLH_firstorder_Range,

wavelet_LLH_firstorder_RobustMeanAbsoluteDeviation,

wavelet_LLH_firstorder_RootMeanSquared,

wavelet_LLH_firstorder_Skewness,

wavelet_LLH_firstorder_TotalEnergy,

wavelet_LLH_firstorder_Uniformity,

wavelet_LLH_firstorder_Variance,

wavelet_LLH_glcm_Autocorrelation,

wavelet_LLH_glcm_ClusterProminence,

wavelet_LLH_glcm_ClusterShade,

wavelet_LLH_glcm_ClusterTendency,

wavelet_LLH_glcm_Contrast,

wavelet_LLH_glcm_Correlation,

wavelet_LLH_glcm_DifferenceAverage,

wavelet_LLH_glcm_DifferenceEntropy,

wavelet_LLH_glcm_DifferenceVariance,

wavelet_LLH_glcm_Id,

wavelet_LLH_glcm_Idm,

wavelet_LLH_glcm_Idmn,

wavelet_LLH_glcm_Idn,

wavelet_LLH_glcm_Imc1,

wavelet_LLH_glcm_Imc2,

wavelet_LLH_glcm_InverseVariance,

wavelet_LLH_glcm_JointAverage,

wavelet_LLH_glcm_JointEnergy,

wavelet_LLH_glcm_JointEntropy,

wavelet_LLH_glcm_MaximumProbability,

wavelet_LLH_glcm_SumEntropy,

wavelet_LLH_glcm_SumSquares,

wavelet_LLH_gldm_DependenceEntropy,

wavelet_LLH_gldm_DependenceNonUniformity,

wavelet_LLH_gldm_DependenceNonUniformityNormalized,

wavelet_LLH_gldm_DependenceVariance,

wavelet_LLH_gldm_GrayLevelNonUniformity,

wavelet_LLH_gldm_GrayLevelVariance,

wavelet_LLH_gldm_HighGrayLevelEmphasis,

wavelet_LLH_gldm_LargeDependenceEmphasis,

wavelet_LLH_gldm_LargeDependenceHighGrayLevelEmphasis,

wavelet_LLH_gldm_LargeDependenceLowGrayLevelEmphasis,

wavelet_LLH_gldm_LowGrayLevelEmphasis,

wavelet_LLH_gldm_SmallDependenceEmphasis,

wavelet_LLH_gldm_SmallDependenceHighGrayLevelEmphasis,

wavelet_LLH_gldm_SmallDependenceLowGrayLevelEmphasis,

wavelet_LLH_glrlm_GrayLevelNonUniformity,

wavelet_LLH_glrlm_GrayLevelNonUniformityNormalized,

wavelet_LLH_glrlm_GrayLevelVariance,

wavelet_LLH_glrlm_HighGrayLevelRunEmphasis,

wavelet_LLH_glrlm_LongRunEmphasis,

wavelet_LLH_glrlm_LongRunHighGrayLevelEmphasis,

wavelet_LLH_glrlm_LongRunLowGrayLevelEmphasis,

wavelet_LLH_glrlm_LowGrayLevelRunEmphasis,

wavelet_LLH_glrlm_RunEntropy,

wavelet_LLH_glrlm_RunLengthNonUniformity,

wavelet_LLH_glrlm_RunLengthNonUniformityNormalized,

wavelet_LLH_glrlm_RunPercentage,

wavelet_LLH_glrlm_RunVariance,

wavelet_LLH_glrlm_ShortRunEmphasis,

wavelet_LLH_glrlm_ShortRunHighGrayLevelEmphasis,

wavelet_LLH_glrlm_ShortRunLowGrayLevelEmphasis,

wavelet_LLH_glszm_GrayLevelNonUniformity,

wavelet_LLH_glszm_GrayLevelNonUniformityNormalized,

wavelet_LLH_glszm_GrayLevelVariance,

wavelet_LLH_glszm_HighGrayLevelZoneEmphasis,

wavelet_LLH_glszm_LargeAreaEmphasis,

wavelet_LLH_glszm_LargeAreaHighGrayLevelEmphasis,

wavelet_LLH_glszm_LargeAreaLowGrayLevelEmphasis,

wavelet_LLH_glszm_LowGrayLevelZoneEmphasis,

wavelet_LLH_glszm_SizeZoneNonUniformity,

wavelet_LLH_glszm_SizeZoneNonUniformityNormalized,

wavelet_LLH_glszm_SmallAreaEmphasis,

wavelet_LLH_glszm_SmallAreaHighGrayLevelEmphasis,

wavelet_LLH_glszm_SmallAreaLowGrayLevelEmphasis,

wavelet_LLH_glszm_ZoneEntropy,

wavelet_LLH_glszm_ZonePercentage,

wavelet_LLH_glszm_ZoneVariance,

wavelet_LLH_ngtdm_Busyness,

wavelet_LLH_ngtdm_Coarseness,

wavelet_LLH_ngtdm_Complexity,

wavelet_LLH_ngtdm_Contrast,

wavelet_LLH_ngtdm_Strength,

wavelet_LLL_firstorder_10Percentile,

wavelet_LLL_firstorder_90Percentile,

wavelet_LLL_firstorder_Energy,

wavelet_LLL_firstorder_Entropy,

wavelet_LLL_firstorder_InterquartileRange,

wavelet_LLL_firstorder_Kurtosis,

wavelet_LLL_firstorder_Maximum,

wavelet_LLL_firstorder_Mean,

wavelet_LLL_firstorder_MeanAbsoluteDeviation,

wavelet_LLL_firstorder_Median,

wavelet_LLL_firstorder_Minimum,

wavelet_LLL_firstorder_Range,

wavelet_LLL_firstorder_RobustMeanAbsoluteDeviation,

wavelet_LLL_firstorder_RootMeanSquared,

wavelet_LLL_firstorder_Skewness,

wavelet_LLL_firstorder_TotalEnergy,

wavelet_LLL_firstorder_Uniformity,

wavelet_LLL_firstorder_Variance,

wavelet_LLL_glcm_Autocorrelation,

wavelet_LLL_glcm_ClusterProminence,

wavelet_LLL_glcm_ClusterShade,

wavelet_LLL_glcm_ClusterTendency,

wavelet_LLL_glcm_Contrast,

wavelet_LLL_glcm_Correlation,

wavelet_LLL_glcm_DifferenceAverage,

wavelet_LLL_glcm_DifferenceEntropy,

wavelet_LLL_glcm_DifferenceVariance,

wavelet_LLL_glcm_Id,

wavelet_LLL_glcm_Idm,

wavelet_LLL_glcm_Idmn,

wavelet_LLL_glcm_Idn,

wavelet_LLL_glcm_Imc1,

wavelet_LLL_glcm_Imc2,

wavelet_LLL_glcm_InverseVariance,

wavelet_LLL_glcm_JointAverage,

wavelet_LLL_glcm_JointEnergy,

wavelet_LLL_glcm_JointEntropy,

wavelet_LLL_glcm_MaximumProbability,

wavelet_LLL_glcm_SumEntropy,

wavelet_LLL_glcm_SumSquares,

wavelet_LLL_gldm_DependenceEntropy,

wavelet_LLL_gldm_DependenceNonUniformity,

wavelet_LLL_gldm_DependenceNonUniformityNormalized,

wavelet_LLL_gldm_DependenceVariance,

wavelet_LLL_gldm_GrayLevelNonUniformity,

wavelet_LLL_gldm_GrayLevelVariance,

wavelet_LLL_gldm_HighGrayLevelEmphasis,

wavelet_LLL_gldm_LargeDependenceEmphasis,

wavelet_LLL_gldm_LargeDependenceHighGrayLevelEmphasis,

wavelet_LLL_gldm_LargeDependenceLowGrayLevelEmphasis,

wavelet_LLL_gldm_LowGrayLevelEmphasis,

wavelet_LLL_gldm_SmallDependenceEmphasis,

wavelet_LLL_gldm_SmallDependenceHighGrayLevelEmphasis,

wavelet_LLL_gldm_SmallDependenceLowGrayLevelEmphasis,

wavelet_LLL_glrlm_GrayLevelNonUniformity,

wavelet_LLL_glrlm_GrayLevelNonUniformityNormalized,

wavelet_LLL_glrlm_GrayLevelVariance,

wavelet_LLL_glrlm_HighGrayLevelRunEmphasis,

wavelet_LLL_glrlm_LongRunEmphasis,

wavelet_LLL_glrlm_LongRunHighGrayLevelEmphasis,

wavelet_LLL_glrlm_LongRunLowGrayLevelEmphasis,

wavelet_LLL_glrlm_LowGrayLevelRunEmphasis,

wavelet_LLL_glrlm_RunEntropy,

wavelet_LLL_glrlm_RunLengthNonUniformity,

wavelet_LLL_glrlm_RunLengthNonUniformityNormalized,

wavelet_LLL_glrlm_RunPercentage,

wavelet_LLL_glrlm_RunVariance,

wavelet_LLL_glrlm_ShortRunEmphasis,

wavelet_LLL_glrlm_ShortRunHighGrayLevelEmphasis,

wavelet_LLL_glrlm_ShortRunLowGrayLevelEmphasis,

wavelet_LLL_glszm_GrayLevelNonUniformity,

wavelet_LLL_glszm_GrayLevelNonUniformityNormalized,

wavelet_LLL_glszm_GrayLevelVariance,

wavelet_LLL_glszm_HighGrayLevelZoneEmphasis,

wavelet_LLL_glszm_LargeAreaEmphasis,

wavelet_LLL_glszm_LargeAreaHighGrayLevelEmphasis,

wavelet_LLL_glszm_LargeAreaLowGrayLevelEmphasis,

wavelet_LLL_glszm_LowGrayLevelZoneEmphasis,

wavelet_LLL_glszm_SizeZoneNonUniformity,

wavelet_LLL_glszm_SizeZoneNonUniformityNormalized,

wavelet_LLL_glszm_SmallAreaEmphasis,

wavelet_LLL_glszm_SmallAreaHighGrayLevelEmphasis,

wavelet_LLL_glszm_SmallAreaLowGrayLevelEmphasis,

wavelet_LLL_glszm_ZoneEntropy,

wavelet_LLL_glszm_ZonePercentage,

wavelet_LLL_glszm_ZoneVariance,

wavelet_LLL_ngtdm_Busyness,

wavelet_LLL_ngtdm_Coarseness,

wavelet_LLL_ngtdm_Complexity,

wavelet_LLL_ngtdm_Contrast,

wavelet_LLL_ngtdm_Strength
